# Lactic acid bacteria isolated from women’ breast milk and infants’ faeces have appreciable immunogenic and probiotic potentials against diarrheagenic *E. coli* strains

**DOI:** 10.1186/s12866-024-03502-2

**Published:** 2024-09-17

**Authors:** Abiola O. Obisesan, Oyindamola O. Abiodun, Funmilola A. Ayeni

**Affiliations:** 1 Department of Pharmaceutical Microbiology, Faculty of Pharmacy, University of Ibadan, Ibadan, Oyo State, Nigeria; 2 Present Address: Department of Pharmaceutical Microbiology and Biotechnology, College of Pharmacy, Afe Babalola University, Ado Ekiti, Nigeria; 3 Department of Pharmacology and Toxicology, Faculty of Pharmacy, University of Ibadan, Ibadan, Oyo State Nigeria; 4 Department of Environmental and Occupational Health, School of Public Health, Indiana University Bloomington, Bloomington, Indiana, USA

**Keywords:** Mothers’ breastmilk, Neonates’ faeces, Lactic acid bacteria, Antimicrobial, Immunomodulatory

## Abstract

Diarrheal diseases remain the leading cause of high mortality among the infants, particularly in the developing countries; Probiotic intervention for diarrhea has been an ongoing novel approach to diarrheal prevention and treatment. This study aims to characterize immunogenic and probiotic properties of lactic acid bacteria (LAB) isolated from human breast milk and neonates’ faeces. The LAB isolates from 16 mothers’ breast milk and 13 infants’ faeces were screened and identified by 16 S rRNA gene partial sequencing. Their antimicrobial activities against 5 strains of diarrheagenic *Escherichia coli* were tested. Organic acids production was quantified by HPLC, and antibiotic resistance pattern were determined by VITEK^®^. Autoaggregation, co-aggregation and hydrophobicity properties were assessed by UV spectrophotometry and immunomodulatory effect was determined in mouse model. Ninety-three LAB of five genera were identified. The most abundant species was *Lactiplantibacillus plantarum* with inhibition zones ranged from 8.0 to 25.0 ± 1 mm. *Lacticaseibacillus rhamnosus* A012 had 76.8 mg/mL lactic acid, (the highest concentration), was susceptible to all antibiotics tested. *L. plantarum* A011 and *L. rhamnosus* A012 were highly resistance to gastrointestinal conditions. *L. rhamnosus* A012 produced hydrophobicity of 25.01% (*n*-hexadecane), 15.4% (xylene) and its autoaggregation was 32.52%. *L. rhamnosus* A012 and *L. plantarum* A011 exert immunomodulatory effects on the cyclophosphamide-treated mice by upregulating anti-inflammatory cytokine and downregulating proinflammatory cytokines. *Lactobacillus* sp. demonstrated good probiotic and immunomodulatory properties. Further works are ongoing on the practical use of the strains.

**Supplementary Information:**

The online version contains supplementary material available at 10.1186/s12866-024-03502-2.

## Introduction

Diarrheal disease is mostly caused by viral, bacterial, and parasitic microorganisms [1] and often refers to as ‘leading killer’ of children under the age of five worldwide [2]. According to UNICEF and WHO report, about 1.7 billion cases yearly was attributed to childhood diarrheal disease worldwide [3] with around 525,000 deaths of children under the age of five. In 2015, 300,000 children death was recorded globally while over 1300 death was recorded each day representing 484,000 deaths of young children in 2019, however, most of these deaths take place majorly in Africa [2–6]. Despite the awareness and management of diarrhea globally, in 2021, UNICEF still recorded high percentage of death due to diarrhea and reported this disease as a leading killer with approximately 9 per cent of all deaths among children below age 5 globally [4]. The updated UNICEF data report in January 2024 recoded 1,200 young children dying each day, amounting to 443,832 children per year, The occurrence of diarrheal disease is normally high in developing countries, India has the highest numbers of diarrhea cases among children and Nigeria follows with the second largest percentage [7, 8]. Moreover, current UNICEF data showed that for every 1000 birth in Nigeria, 107.2 deaths are recorded annually [4], in which high proportion of these deaths are recorded from Northern Nigeria [9].

Many microorganisms are implicated in diarrhea disease, these include rotavirus, bacteria of different species such as *Vibrio cholerae*,* Campylobacter jejuni*,* Shigella* spp., *Cryptosporidium* sp., *Salmonella* spp., and enteric pathogenic *Escherichia coli* strains of seven pathotypes which include Adherent-Invasive *E. coli* (AIEC), enteropathogenic *E. coli* (EPEC), shiga toxin–producing *E. coli* (STEC), enteroaggregative *E. coli* (EAEC), enteroinvasive *E. coli* (EIEC), enterotoxigenic *E. coli* (ETEC), and diffusely adherent *E. coli* (DAEC) [10, 11]. These pathotypes are pathogenic, they often express virulence factor encoding genes which are involved in activities such as invasion, adhesion, attachment, toxins released and motility which are deficient in natural gastrointestinal *E. coli* [10, 12, 13]. They are, therefore, the principal cause of both acute and severe cause of diarrhea in children.

Most often, antibiotics and Oral Rehydration Therapy (ORT) are employed as a direct approach to combat acute and severe Diarrheagenic *E. coli* (DEC) diseases in children [14–16]. However, these interventions are not sufficient considering the inconsistence in pathogenicity of diarrhea and frequent use of antibiotics disrupts gastrointestinal microbiota [17]. Therefore, a non-antibiotic intervention such as probiotics and fermented foods are encouraged in the management of infantile diarrhea caused by DEC.

Probiotics are beneficial living microorganisms with significant health benefits. They positively affect gut microflora by contributing to intestinal microbial balance [18], general antimicrobial properties and decreasing the harmful microbial toxic activity [19, 20]. They also restore and maintain the gut microbial niche [21]. Probiotic strains possess the potential to reverse the infectious effect due to diarrheagenic microorganisms. The mechanisms of interaction between gut microbial populations and the host immune system reveal that these beneficial microorganisms stimulate and regulate the nerve, endocrine, and immune cells [22]. They also modulate series of activities like digestion, metabolism, competitive removal of pathogens, and neural plasticity [23].

Breastmilk is safe, clean, and best food with necessary nutrients given to infants in their first months of life to promote a sound health and rapid growth [24]. It contains antibody that protects the infants against common illness and infections. According to Moles et al. [24] and Timmerman et al. [25], breastmilk is one of the factors that contributes to the colonization of baby’s gut microbiota as it contains some beneficial microbes such as *Lactobacillus*, *Streptococcus*, *Bifidobacterium*,* Enterobacterium*,* Enterococcus*, *Staphylococcus* among others. Breastmilk microbiota on the other hand influence the colonization and proliferation of the neonate’s gut microbiota and the maturation of immune system. These gut bacteria display their mechanism of actions against gastroenteritis through bidirectional interactions such as direct microbiota-microorganism interactions or indirectly through modulating the host nerve cells and immune cells [22, 26].

A stable microbial community is highly needed to encourage host-microbial and microbial-microbial interaction to improve good health condition among children [27, 28]. Beneficial microbes e.g. lactic acid bacteria (LAB) have been shown to have antibacterial activities against pathogens implicated in gastroenteritis [29–31]. *Lactobacillus* and *Bifidobacterium* are added to baby formula to promote a balanced gut microbiota and prevents the growth of harmful bacteria that could cause infections and inflammation [32]. Currently, there is scarcity of information on LAB from healthy infants’ gut and humans’ breast milk as potential probiotic strains against DEC strains in a diarrhea endemic region. Therefore, this study was designed to characterize and evaluate probiotic potentials of LAB isolated from human mothers’ breastmilk and their infants’ faeces in the management of infantile DEC.

## Methods

### Samples collection

The study was ethically reviewed and approved by the ethics committee of the Ekiti State University Teaching Hospital Ethics and Research Committee and was registered as EKSUTH/A67/2015/03/008. Prior to the collection of samples, informed consent of the participants (mother and child) was obtained from the mothers. Fresh mothers’ breastmilk and infants’ faecal samples were collected from the participants (nursing mothers and children) at the Department of Community Medicine, Ekiti State University Teaching Hospital, Ado Ekiti, Ekiti State, Nigeria. A total of 16 breastmilk samples and 13 fecal samples were collected from 16 mothers and 13 infants. The age of the mothers ranged between 23 years to 33 years while their neonates age ranged between 1 day to 9 months old. The collection of samples was done by health personnel from the Community Medicine Unit of the hospital in an aseptic environment. Nipples and areola of the mothers’ breast were cleaned properly with soap and sterile water before manually expressing the breast milk, the first three drops of breastmilk were discarded. Five mL of breastmilk samples was collected from mothers into sterile collection tubes. Approximately 6–10 g of fresh fecal samples were also collected from the neonates into sterile collection tubes. The samples collected were stored on ice until delivery to the laboratory for immediate microbiological analysis. The test pathogenic microorganisms used for the study were 5 diarrheagenic *E. coli* strains; EPEC H62E, ETEC H40B, STEC H77E, EIEC H68D, and EAEC H40C obtained from Molecular and Biotechnology laboratory, Pharmaceutical Microbiology Department, University of Ibadan, Nigeria.

### Isolation of lactic acid bacteria

The isolation of LAB from the samples was carried out using modified method of Medjaoui et al. [33]. In summary, 1 mL of breastmilk sample/1 g of faecal sample was homogenized with 9 mL of de Man–Rogosa and Sharpe broth (Oxoid, U.K) supplemented with 0.05 mg/100 mL of L-cysteine hydrochloride (MERCK, Darmstadt, Germany), (MRS-cys) to make a 10^− 1^ dilution. The mixture was incubated at 37^o^C under anaerobic condition (AnaerogenGen^TM^Oxoid, UK) for 24 h. The resulting cultures were homogenized for 1 min, ten-fold serial dilutions were done and 100 µL of last 3 dilutions was plated out on MRS-cys agar for 48 h. Morphologically distinct colonies were sub-cultured onto MRS-cys agar plates until pure colony was achieved. Gram positive and catalase negative LAB isolates were preserved and stored as glycerol/MRS-cys stocks at − 80 °C.

### Identification of lactic acid bacterial strains

The Genomic DNA of LAB was extracted from overnight broth culture using *AccuPrep*^®^ Genomic DNA Extraction kit (Bioneer, South Korea), according to the manufacturer`s instructions. The extracted DNA was used in a PCR reaction with 27 F (AGAGTTTGATCMTGGCTCAG) and 1389R (ACGGGCGGTGTGTACAAG) primers. The PCR reaction was an initial denaturation at 95 °C for 4 min followed by 25 cycles of 95 °C for 1 min, 55 °C for 1 min and 72 °C for 1 min 30 s and a final 7 min extension at 72 °C [34]. The amplicons obtained were stained using Gel-Red, analyzed by agarose gels electrophoresis (1.5% w/v agarose), and their sizes were visualized with Gel documentation system (E-Box CX5.TS.26MX Trans-illuminator System, VILBER, France). The nucleotides sequences obtained were analyzed using The Basic Local Alignment Search Tool (BLAST) at http://www.ncbi.nlm.nih.gov/BLAST. and the identified sequences were placed in the European Molecular Biology Laboratory (EMBL) with accession number PRJNA628165 (https://www.ncbi.nlm.nih.gov/sra/PRJNA628165).

### Determination of antimicrobial activities of isolated strains

To determine the anti-diarrheagenic *E. coli* activities of isolated LAB, agar well diffusion method was used [35]. Briefly, fresh LAB isolates were cultured for 24 h at 37 °C, centrifuged for 10 min at 12,000 rpm, and sterilized by filtration through a 0.45 μm filter (Millipore, Billerica, MA, United States). A 0.1 mL of 24 h fresh cultured of each diarrheagenic *E. coli* strain (10^6^ cfu/mL) was seeded onto a sterile petri dish containing pre-solidified Mueller Hinton agar (Becton, Dickson and Co, Spark, MD, USA) and allowed to dry. The equidistant 6 mm diameter wells were made with a sterile borer. An aliquot of 100 µL of the cell free supernatant (CFS) was dispensed into each well bored and was allowed to diffuse at room temperature for 60 min. The plates were incubated for 24 h at 37^o^C. The antimicrobial activity of the LAB indicated by zones of inhibitions surrounding each well containing CFS were measured and recorded. All the tests were performed in triplicates.

The agar overlay method described by Ayeni et al. [19] was employed and modified to determine the activity of viable LAB cells against the 5 diarrheagenic *E. coli* strains. Briefly, a fresh cultured LAB isolate was prepared overnight in MRS broth culture, 50 µL of LAB was added on MRS-cys agar plates as a straight line and incubated anaerobically for 48 h at 37^o^C. Thereafter, the agar plates were overlaid with a fresh broth culture of each diarrheagenic *E. coli* (10^6^ cfu/mL) vehiculated in 10 mL Mueller Hinton soft agar (0.7%). The overlay agar was left to set and incubated aerobically for 24 h at 37^o^C. The zone of inhibition around the straight LAB lines were measured. All the assays were conducted in triplicate.

### Growth inhibition of pathogenic strains by LAB using co-culture assay

The growth inhibition of diarrheagenic *E. coli* strains by selected LAB isolates (they were previously selected due to good antimicrobial activities results) were carried out in a kinetic study. Ten (10) ml MRS-MH broth that contained 5 ml of MRS-cys (double strength) and 5 ml of Mueller Hinton broths (double strength) were prepared to allow the respective growth of LAB and *E. coli*. The co-culture broth was inoculated with 10^8^ cfu/mL of each of 15 *Lactobacillus* isolates, and 10^6^ cfu/mL of each of the 5 diarrheagenic *E. coli* strains and incubated at 37 ^o^C for 24 h. Each *Lactobacillus* isolate and diarrheagenic *E. coli* strain were mono-cultured and used as the control. Viable counts of *E. coli* and LAB strains in co-culture tubes were done at 0, 8, 16, 24 h by plating them onto sterile Eosin Methylene Blue agar plates (Sigma-Aldrich, USA) and sterile MRS-cys agar plates for the determination of viable cell. The plates were incubated for 24 h at 37 ^o^C [36, 37].

One of the major mechanisms of antimicrobial activity of LAB is production of organic acid, therefore, LAB isolates were analyzed for organic acid productions. The organic acids, which include propionic, acetic, lactic, and butyric acids were measured and quantified by HPLC (Adept CECIL CE 4200). The HPLC procedure was carried out using 20 µL of the supernatant LAB isolates. The supernatant was pipette directly into the HPLC system fitted to the UV absorbance detector with 210 nm wavelength. H_2_SO_4_ at 55^o^C was the mobile phase and the HPLC standard curves were plotted using the retention time data and response factor of the organic acids. Then, the area (mAs), height (mA) and the quantity of organic acids (mg/mL) produced by the isolates were measured with linear coefficients (R^2^) greater than 0.99 [37].

### Resistance of LAB strains to gastrointestinal conditions

#### Resistance to low pH

The acid tolerance of the LAB isolates at different pH level was assessed using the modified method of Hassanzadazar et al. [38]. Overnight cultures of LAB were centrifuged at 12,000 rpm for 10 min. The supernatants were discarded, and the pellets were carefully rinsed twice with phosphate buffer saline (PBS; 7.2). The cell pellets were re-suspended in 10 mL of adjusted pH 2.0 and 3.0 MRS-cys broth and incubated for 3 h at 37^o^C. The survived bacterial cells at 0 and 3 h were determined by plating out 1 mL of the broth and incubating at 37^o^C for 24 h, the colonies were counted using colony counter. The LAB isolates cultured without any adjustment of pH were used as control. Each assay was conducted in triplicate.

### Tolerance of LAB to bile salt

The tolerance of LAB to bile salt was determined by a modified method of Hassanzadazar et al. [38]. Overnight cultures of LAB isolates were freshly prepared in MRS-cys broth at 37 °C, the prepared cultures were centrifuged at 12,000 rpm for 10 min. The supernatants were discarded, and the cell pellets were gently rinsed twice in 7.2 pH phosphate buffer saline, re-suspended in a 10 ml sterile MRS-cys broth supplemented in 0.3% (w/v) bile salt and incubated at 37 °C for 4 h under anaerobic condition (AnaeroGen™ 3.5 L). The survived bacterial cells at 0 and 4 h were determined by plating out 1 mL of the broth and incubating at 37^o^C for 24 h, the colonies were counted using colony counter. The LAB isolates cultured without any adjustment of pH was used as control. Each assay was conducted in triplicate.

.

### Consecutive acid and bile tolerance test

The ability of LAB isolates to survive gastrointestinal transit of consecutive low pH and bile supplementation was evaluated for the selected 20 LAB isolates with reference to their antibacterial potential and tolerance to mono bile and acid environment. Freshly prepared LAB broth cultures were centrifuged at 12,000 rpm for 10 min. The bacterial supernatants were discarded, and cells pellets were gently rinsed twice in 7.2 pH phosphate buffer saline and re-suspended in a 10 mL sterile MRS-cys broth. An aliquot of 0.1 mL of the suspension was re-introduced into 10 mL MRS broth adjusted to pH 3 with 1 M HCl (the initial viable counts were determined by plating out and incubating 1 mL of the mixture) and then the tubes were incubated for 3 h at 37^o^C under anaerobic condition, thereafter, 0.1 ml of the mixture was further inoculated into 10 ml of MRS-cys broth of 0.3% bile salt (w/v), and incubated at 37^o^C for 4 h under anaerobic condition. The final survived bacterial cells at 4 h were determined by plating out 1 mL of the broth and incubating at 37^o^C for 24 h, The LAB isolates cultured without any adjustment, nor any supplementation were used as control. The log reduction of final count in comparison with the initial count was determined. Each experiment was conducted in triplicate.

### Cell surface hydrophobicity

Cell surface hydrophobicity is an important factor of Microbial Adhesion to Hydrocarbon (MATH), it is employed to determine the contact of bacteria with their host cells [39]. Based on this, ninety-three strains of LAB were evaluated for cell surface hydrophobicity using a previously described method [36] with modification. A fresh overnight LAB broth culture was prepared and centrifuged (5,000 rpm for 15 min). to obtain the cell pellet. LAB cells were washed two times with sterile PBS. The re-suspended sterile PBS (3mL) and the optical densities was adjusted to 600 nm absorbance for initial reading with the use of UV spectrophotometer (Unico, Flinn Scientific, Canada). Then, 1 mL each of two hydrocarbons; xylene (Sigma, USA) and *n*-hexadecane (Sigma, USA) was pipetted and added to the suspension. The tubes were vortexed vigorously for 30 s, incubated for 1 h at 37 °C. The aqueous phase was carefully collected and its absorbance at 600 nm was measured. The result was expressed in percentage. Cell surface hydrophobicity was calculated using the equation below:

$$\eqalign{& \% {\rm{ Hydrophobicity}}\, \cr & = \,{{\left[ {\scriptstyle \left[ {{\rm{Optical}}\,{\rm{Density}}\,{\rm{initial}}\,{\rm{absorbance }}\left( {600{\rm{nm}}} \right)} \right. \hfill \atop \scriptstyle -{\rm{ O}}{\rm{.D}}{\rm{.}}\,{\rm{final}}\,{\rm{absorbanc}}e\,\left( {600nm} \right) \hfill} \right]} \over {{\rm{Optical}}\,{\rm{Density}}\,{\rm{initial}}\,{\rm{absorbance}}\,{\rm{ }}\left( {600{\rm{nm}}} \right)}}\, \cr & \times 100 \cr}$$

### Autoaggregation and Co-aggregation assay

Auto-aggregation is used most often as a pre-test to evaluate the adhesion property of the LAB to mucosal and epithelial surfaces [39]. This assay is used to evaluate aggregation of bacteria cell, based on the ability of LAB isolates to survive the gastric-intestinal environment and their adherence to hydrocarbon, the auto-aggregation of 15 potential probiotic LAB isolates (they were previously selected due to good gastrointestinal tolerance results) was evaluated using previously described method [40] with some modification. A fresh overnight LAB broth culture was prepared and centrifuged (5,000 rpm for 15 min) to obtain the cell pellet. LAB cells were washed two times with sterile PBS. The re-suspended sterile PBS (3 mL) was adjusted to 600 nm absorbance for initial reading with the use of UV spectrophotometer (Unico, Flinn Scientific, Canada). Thereafter, the remaining suspension was also vortexed and left to incubate for 5 h at 37^o^C. At an hour interval up to 5 h (t1, t2, t3, t4 and t5), an aliquot (1 mL) from the top of the suspension was carefully removed and its absorbance at 600 nm was measured and recorded. Autoaggregation result was determined using the following equation:

$$\% {\rm{ Autoaggregation}}\,{\rm{ = }}\,{{\left[ {\left( {{{\rm{A}}_0} - {{\rm{A}}_{\rm{t}}}} \right){\rm{ }} \times {\rm{ }}100} \right]} \over {{A_0}}}$$

Note A_0_ indicates the absorbance at time 0 h.

A_t_ indicates the absorbance every hour, up to 5 h.

To determine the co-aggregation potential of LAB, the modified method of Pessoa et al. [40] was used. Briefly, cell suspensions of selected LAB and 5 diarrheagenic *E. coli* strains were prepared. Then, 2 mL of mixed suspensions (1 ml of LAB cell suspension and 1 mL diarrheagenic *E. coli*) were vortex and incubated for 5 h at 37^o^C co-cultured. Also, 2 ml of each (LAB cell suspension and diarrheagenic *E. coli* strains cell suspensions) were incubated at the same conditions (control). The absorbance at 600 nm wavelength of co-cultured and mono-cultured suspensions were measured using UV spectrophotometer (Chongqing Gold, China). The co-aggregation in % was calculated as written below:

$$\eqalign{& Coaggregation\,\left( \% \right) \cr & = \,\left[ {\left( {Ax\, + \,Ay} \right)/2\,-\,A\left( {x + y} \right)} \right]/\left( {Ax\, + \,Ay} \right)/2] \cr}$$

Ax and Ay represents absorbance of LAB and diarrheagenic *E coli* strains separately.

A(x + y) represents the absorbance of mixed culture of LAB suspensions and diarrheagenic *E coli* suspensions.

### Evaluation of antibiofilm potential of LAB

Due to the protective properties of LAB against pathogens and their associated biofilms, fifteen selected LAB that previously produced efficient inhibition against EAEC H40C was analysed to produce antibiofilm agents against a characterized EAEC 042 strain [41] using modified method of Jadhav et al. [42]. Three dilutions of CSF of LAB cells (1:1, 1:10 and 1:100) were prepared. 180 µl of high glucose Dulbecco′s Modified Eagle′s Medium (DMEM) broth (Sigma Aldrich, USA), was placed into each 96-well flat bottom polystyrene plates together with 15 µl of CFS of LAB and 5 µl of EAEC 042 culture to make a total of 200 µl suspensions. A high glucose DMEM broth with no EAEC 042 was used as control. Absorption of 595 nm OD was measured for initial growth inhibition; the sealed plates were aerobically incubated for 18 h at 37^0^C. The plates were washed thoroughly using 200 µL of sterile PBS in a microplate washer (Global diagnostics Micro wash 1100) thrice, the air-dried plate was fixed for 10 min using 75% ethanol. The plate was stained for 5 min with 0.5% crystal violet, then, rinsed to remove the unbound dye. The plate was dried and eluted using 200 µL of 95% ethanol for 20 min.

The biofilm produced was quantified using microplate spectrophotometer (BioRad, Richmond, CA, USA), the optical density of the eluted crystal violet was measured at 570 nm. The amount of the stain absorbed determined the quantity of the biofilm formed.

The biofilm inhibition was expressed in percentage and was calculated using the following formula:

$$\eqalign{& \% \,{\rm{Biofilm}}\,{\rm{inhibition}} \cr & = \,{{{\rm{OD}}570\,{\rm{nm}}\,{\rm{of}}\,{\rm{control}}\, - \,{\rm{OD}}570\,{\rm{nm}}\,{\rm{of}}\,{\rm{test}}} \over {{\rm{OD}}570\,{\rm{nm}}\,{\rm{of}}\,{\rm{control}}}} \cr & \times \,100 \cr}$$

### Evaluation of antibiotics susceptibility pattern of selected lactic acid bacteria

The minimum inhibitory concentration (MIC) of overnight cultures of 15 selected potential probiotic LAB isolates was determined using AST-GP75 test cards with Vitek 2 (Biomerieux diagnostics, France) equipment according to the manufacturers` instructions. The MIC results were interpreted as resistant or susceptible according to European Food Safety Authority (EFSA) [43, 44] breakpoint guidelines.

### Haemolytic pattern of lactic acid bacteria

The haemolytic potential of all LAB isolates was observed using a previously described method [45]. Fresh LAB were streaked on blood agar supplemented with 5% human blood and incubated at 37^o^C for 24 h. Haemolytic properties such as β-, α- and γ-haemolysis production were observed. LAB isolates with γ-haemolysis were recorded as non- haemolytic.

### In-vivo stimulation of immune markers by selected lactic acid bacteria

#### Experimental animals

The use of animals for experimental purpose was approved by Afe Babalola University Ethical Committee with the approved reference number AB/EC/19/06/047. The procedures involving the careful use and proper handling of animals was judiciously followed. Male Swiss mice (5 wk) of 22 ± 4 g weight was obtained from Animal Breeding Experimental Center, Ekiti State University Nigeria and housed in clean ventilated cages with appropriate animal house condition (12/12 light, temperature, humidity). The mice were allowed to acclimatize to their new environment for 1 week and fed with right proportions of laboratory mice pellets and water *ad libitum*. During the study, the weight of the mice were monitored every 4 days with a table top weighing balance, this was done till the end of the experiment.

### Preparation of bacterial strains

Freshly prepared *L. plantarum* A011 and *L. rhamnosus* A012 were cultured using MRS-cys at 37^o^C for 20 h in anaerobic condition. 1.0 × 10^8^ cfu/mL of the cells were harvested, centrifuged for 10 min at 4,000 rpm using compact centrifuge (Biocompare, USA). The cell pellets were rinsed two times, re-suspended in 10 mL of sterile PBS. The suspensions were prepared daily for oral use.

### Experimental design

After a week of acclimatization, the mice were initially divided into seven groups of six mice per group. The first four groups were administered with an immunosuppressive agent, cyclophosphamide (CTX), and were classified as immunosuppressed group, the next two groups were given *L. rhamnosus* A012 and *L. plantarum* A011 respectively, they were classified as immunocompetent group, and the last group was given PBS and was classified as normal group. The detailed of the treatment in each group is as follows: group 1 (CTX + PBS) received cyclophosphamide 80 mg/kg BW/d via intraperitoneal injection (i.p) for three days, thereafter, received PBS (vehicle) for 15 days. Group 2 (CTX + Lev.) received cyclophosphamide 80 mg/kg BW/d via i.p. for three days and treated orally with a standard drug levamisole hydrochloride 40 mg/kg for 15 days. Group 3 (CTX + *L. rham*) received cyclophosphamide 80 mg/kg BW/d via i.p. for three days, thereafter, treated orally with 0.2 ml of *L. rhamnosus* A012 (1.0 × 10^8^ cfu/mL) for 15 days. Group 4 (CTX + *L. pla*) received cyclophosphamide 80 mg/kg BW/d via i.p. for three days and treated orally with 0.2 ml of *L. plantarum* A011 (1.0 × 10^8^ cfu/mL) for 15 days, Group 5 (*L. rham* alone) received 0.2 ml of *L. rhamnosus* A012 (1.0 × 10^8^ cfu/mL) orally for 18 days. Goup 6 (*L. pla* alone) received 0.2 ml of *L. plantarum* A011 (1.0 × 10^8^ cfu/mL) orally for 18 days and group 7 (PBS alone) received 0.2 ml of PBS orally for 18 days.

### Analysis of immune organ index and white blood cells quantification

As described above, the study was carried out for 18 days. The experimental mice were euthanized by inhalation of phentermine hydrochloride at 0.1–0.2 ml depending on the body weight of the animal to induce loss of consciousness. The blood of each mice was collected from abdominal aorta and dispensed immediately in EDTA bottle for whole blood analysis and sterile sample bottle to prepare serum. Cervical dislocation was carried out, the colon and the spleen were harvested and placed immediately in PBS, then refrigerated for ELISA analysis. The spleen index was calculated as follows:

$$\eqalign{& {\rm{Spleen}}\,{\rm{or}}\,{\rm{thymus}}\,{\rm{indices}}\,\left( {mg/g} \right) \cr & = \,{{{\rm{spleen}}\,{\rm{or}}\,{\rm{thymus}}\,{\rm{weight}}\,\left( {{\rm{mg}}} \right)} \over {{\rm{Bodyweight}}\,\left( {\rm{g}} \right)}} \cr}$$

The white blood cells in the whole blood previously collected were microscopically counted with haemocytometer. and recorded as number of cells/ µL.

### Cytokine quantitation

The serum and the spleen concentrations of TNF-α, IL-6, and IL-10 were determined using ELISA kit (LegendMax™, BioLegend, U.K) at room temperature according to the manufacturers` instructions. The quantity of pro- and anti- inflammatory cytokines produced were measured using microplate reader at 450 nm. The concentration of each cytokine was extrapolated using line of regression from the standard curves of TNF-α, IL-6, and IL-10 and expressed as pg /mL.

### Statistical analysis

Graph pad prism 5 and 8.0 statistical software program was used to analyze the results. The statistical significance of the data was determined with one-way ANOVA and the p values less than 0.05.

## Results

### Diversity of lactic acid bacteria in human breast milk and infant faeces

**Fig. 1:**
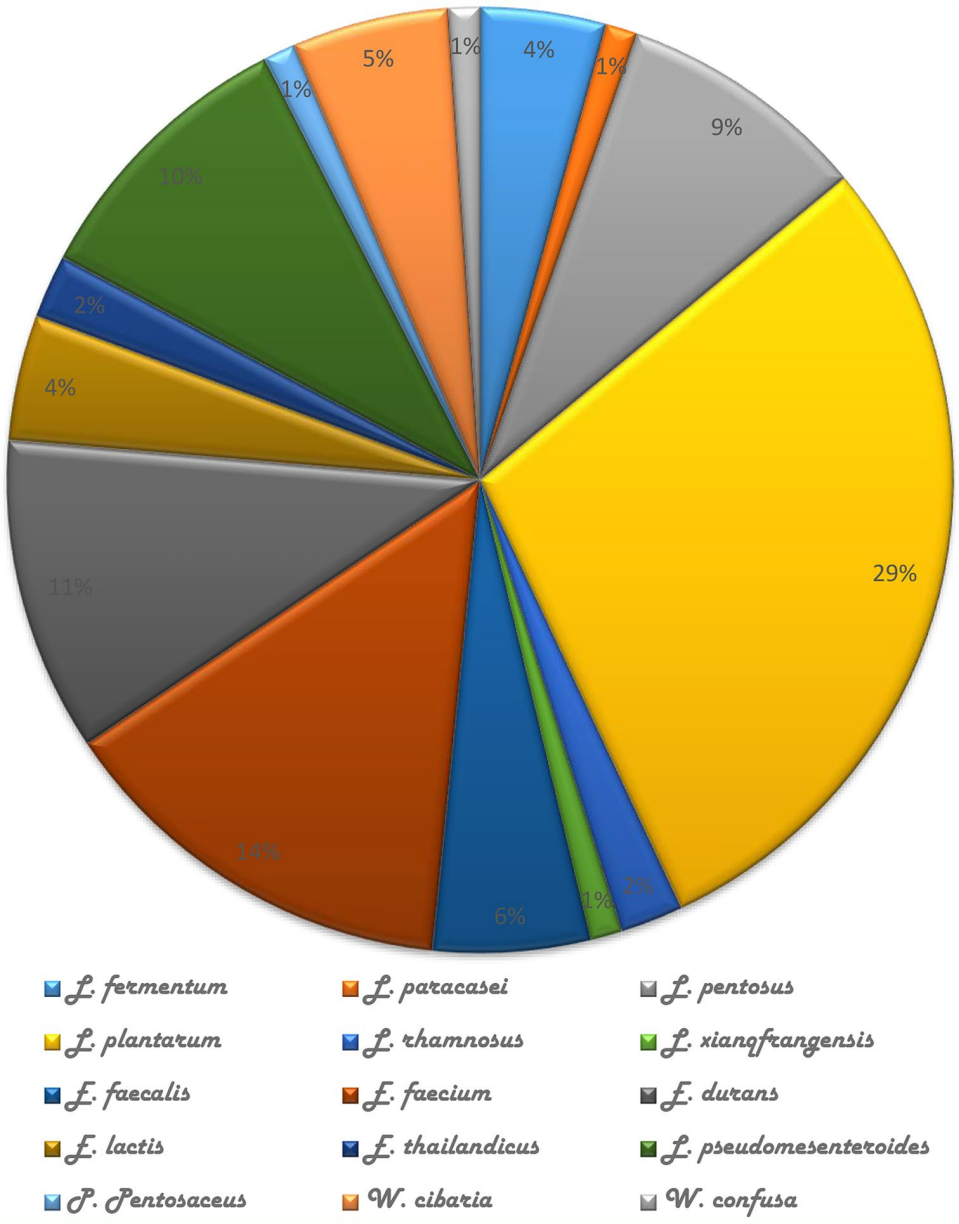
Distribution (%) of isolated LAB from breast milk and faeces

Ninety-three LAB strains were identified from the 2 samples (i.e. breast milk and neonates’ faeces). Five genera of LAB were identified viz.: *Enterococcus*, *Lactobacillus*, *Leuconostoc*,* Weisella*, and *Pediococcus*. The predominant genus was *Lactobacillus* spp. (46.24%), *Pediococcus* spp (1.08%) was the least, the genera comprise of 15 species which include 12 strains of *Enterococcus. faecium*, 4 *Limosilactobacillus. fermentum* strains, 12 *L. pentosus* strains, 27 *L. plantarum* strains, 9 *E. durans* strains, 9 *Leuconostoc. pseudomesenteroides* strains, 7 *E. faecalis* strains, 4 *E. lactis* strains, 5 *Weissella cibaria* strains, 2 *E. thailadicus* strains, 2 *L. rhamnosus* strains, 1 *L. paracasei* strain, 1 *L. xiangfangensis* strain, 1 *W. confusa* and 1 *Pediococcus pentosaceus* strain. (Fig. 1).

### Lactic acid bacteria inhibited the growth of diarrheagenic *E. coli.*

The CFS of LAB exhibited zones of inhibitions against diarrheagenic *E. coli* strains. Out of 93 LAB isolates tested, 34 strains produced zones of inhibition against all strains of diarrheagenic *E. coli* tested, meanwhile, 64 LAB isolates (68.82%) produced zones of inhibition against EPEC H62E, of which *E. faecium* A039 had the highest zone of 18.07 ± 0.06 mm (+++). On the other hand, 57 LAB isolates (61.29%) produced zones of inhibition against EIEC H68D, the highest zone was produced by *L. pentosus* B1b. with 26.1 ± 0.56 mm (++++). In addition, 81 strains of LAB (87.10%) had zones of inhibition against STEC H77E of which *L. pentosus* B1b produced the highest zone with 26.1 ± 0.1 mm (++++). In addition, 84 (90.32%) LAB isolates had zones of inhibition against ETEC H40B in which *L. pseudomesenteroides* A093 produced the highest with 26.07 ± 0.12 mm (++++). Sixty-nine isolates (74.20%) of LAB inhibited the growth of EAEC H40C, the highest zones were produced by *P. pentosaceus* A074 and *L. plantarum* A059 with 23.07 ± 0.06 and 23.4 ± 0.61 mm (++++) (Table 1 and Supplementary Table 1).

**Table 1:** Antibacterial activity of lactic acid bacteria against diarrheagenic *Escherichia coli* strains

LAB species	No of isolates (%)	Zones of inhibition (mm)																			
		ETEC (H40B)				EPEC (H62D)				EIEC (H68D)				STEC (H77E)				EAEC (H40C)			
		+	++	+++	++++	+	++	+++	++++	+	++	+++	++++	+	++	+++	++++	+	++	+++	++++
*L. fermentum*	4(4.%)	1 (1)	0 (1)	0 (1)	0 (0)	0 (1)	1 (0)	0 (3)	0 (0)	3 (3)	1(0)	0 (0)	0 (0)	2 (0)	2 (1)	0 (2)	0 (0)	2 (0)	0 (2)	0 (0)	0 (0)
*L. paracasei*	1(1%)	0 (0)	0 (0)	1 (0)	0 (1)	0 (0)	0 (0)	0 (0)	0 (1)	0 (1)	0 (0)	0 (0)	0 (0)	0 (0)	1 (0)	0 (0)	0 (1)	0 (0)	1 (0)	0 (0)	0 (0)
*L. pentosus*	8 (10%)	4 (3)	2 (2)	1 (3)	0 (0)	3 (2)	2 (0)	1 (2)	0 (2)	3 (0)	1 (3)	1 (2)	1 (0)	1 (1)	2 (2)	3 (3)	0 (1)	0 (2)	6 (3)	0 (0)	0 (0)
*L. plantarum*	27(29%)	2 (2)	17 (3)	4 (10)	3 (12)	9 (3)	8 (5)	3 (8)	0 (11)	11 (6)	5 (10)	1 (5)	0 (2)	5 (2)	8 (2)	9 (9)	4 (14)	8 (2)	8 (2)	10 (11)	1 (3)
*L. rhamnosus*	2(2%)	0 (0)	0 (0)	0 (2)	2 (0)	1(0)	0 (0)	1 (0)	0 (2)	1 (0)	1 (1)	0 (1)	0 (0)	0 (0)	0 (0)	2 (2)	0 (0)	0 (0)	0 (0)	2 (2)	0 (0)
*L. xianqfrangensis*	1(1%)	0 (0)	0 (0)	0 (0)	0 (1)	1(0)	0 (0)	0 (0)	0 (1)	1 (0)	0 (0)	0 (0)	0 (1)	0 (0)	0 (0)	0 (0)	0 (1)	1 (0)	0 (0)	0 (1)	0 (0)
*E. faecalis*	7 (8%)	0 (0)	2 (0)	1 (2)	1 (5)	1(0)	1 (2)	0 (2)	0 (2)	2 (1)	1 (2)	0 (3)	0 (2)	1 (0)	1 (0)	1 (2)	1 (5)	0 (1)	1 (3)	1 (2)	2 (1)
*E. faecium*	12(13%)	1 (1)	4 (1)	4 (4)	3 (6)	4 (1)	5 (1)	1 (6)	0 (2)	7 (1)	1 (1)	0 (2)	0 (0)	0 (1)	4 (2)	4 (1)	4 (7)	0 (2)	5 (1)	5 (4)	2 (0)
*E. durans*	9 (10%)	2 (0)	1 (2)	5 (3)	2 (3)	4 (0)	4 (1)	2 (3)	0 (2)	5 (1)	1 (2)	0 (2)	0 (0)	0 (1)	5 (0)	5 (2)	0 (6)	1 (2)	5 (2)	2 (1)	2 (3)
*E. lactis*	4(4%)	1 (0)	2 (0)	0 (3)	1 (1)	0 (0)	2 (1)	0 (2)	0 (1)	0 (0)	0 (2)	0 (1)	0 (1)	0 (0)	2 (0)	1 (1)	1 (3)	0 (1)	2 (1)	1 (1)	1 (0)
*E. thailadicus*	2 (2%)	2 (0)	0 (0)	0 (0)	0 (2)	0 (0)	1 (1)	0 (1)	0 (0)	1 (1)	0 (0)	0 (0)	0 (0)	0 (0)	2 (0)	0 (0)	0 (2)	0 (0)	1 (0)	0 (2)	1 (0)
*L. pseudomesenteroides*	9 (10%)	0 (0)	4(2)	2 (1)	3 (5)	2 (1)	4 (2)	0 (2)	0 (3)	3 (2)	3 (3)	0 (2)	0 (1)	1 (0)	4 (3)	2 (2)	1 (3)	2 (0)	2 (4)	4 (0)	1 (2)
*P. Pentosac*	1(1%)	0 (0)	0 (0)	0 (0)	1 (1)	0 (0)	1 (0)	0 (1)	0 (0)	1 (0)	0 (0)	0 (1)	0 (0)	0 (0)	0 (0)	0 (1)	1 (0)	0 (0)	0 (0)	0 (1)	1 (0)
*W. cibaria*	5(5%)	3 (2)	1 (0)	0 (2)	0 (0)	2 (1)	0 (2)	0 (1)	0 (1)	1 (1)	1 (2)	0 (2)	0 (0)	0 (2)	1 (2)	0 (0)	0 (1)	3 (1)	0 (1)	0 (2)	0 (0)
*W. confuse*	1(1%)	1 (1)	0 (0)	0 (0)	0 (0)	0 (0)	0 (0)	0 (0)	0 (0)	0 (1)	0 (0)	0 (0)	0 (0)	0 (1)	0 (0)	0 (0)	0 (0)	0 (0)	0 (0)	0 (0)	0 (0)

Diameter of zone of inhibition: 8–12 mm = +, > 12–16 mm= ++, ˃16–20 mm = +++, > 20 mm = ++++. The results of viable cells assay are shown in parenthesis

The viable cells of LAB induced large zones of inhibition against the tested pathogens (Table 1). Fourteen LAB isolates produced antimicrobial activities against all tested diarrheagenic *E. coli* strains. Eighty-one (87.10%) strains of LAB produced antimicrobial activity against EPEC H62E, *E. faecalis* A077 produced the largest zone that is designated as ++++ (> 20 mm), 91.40% LAB produced zones of inhibition against ETEC H40B with *E. durans* A004 having the highest zone that is > 20 mm. 76.34% LAB produced zones of inhibition against EAEC H40C in which *E. durans* A098 produced the highest zone that is > 20 mm, 73.12% LAB produced antimicrobial activity against EIEC H68D, *L. pseudomesenteroides* A082 had the highest zone that is > 20 mm. 93.55% LAB produced antagonistic effect against STEC H77E, *L. pseudomesenteroides* A019 produced the highest inhibition that is > 20 mm.

**Fig. 2:**
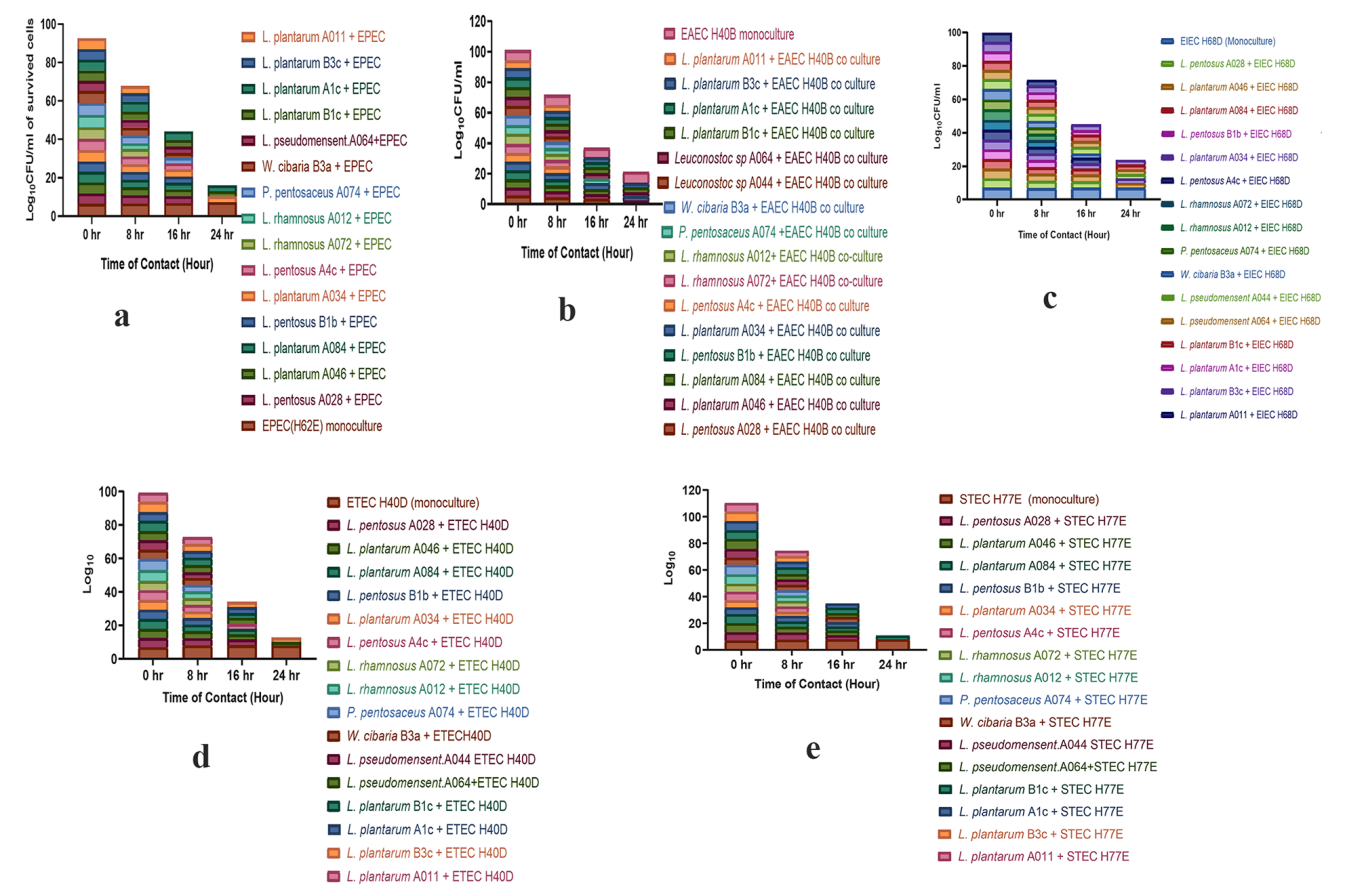
a—e: Kinetic inhibition of diarrheagenic *E. coli* by LAB in co-culture. *Note***a** - LAB isolates vs enteropathogenic E. coli (EPEC) H62E. **b** - LAB isolates vs enteroaggregative E. coli (EAEC) H40B. **c** - LAB isolates vs enteroinvasive *E. coli* (EIEC) H68D. **d** - LAB isolates vs enterotoxigenic *E. coli* (ETEC) H40D. **e** - LAB isolates vs Shiga toxin E. coli (STEC) H62E

The inhibition of growth of 17 LAB against diarrheagenic *E. coli* strains in co-culture experiment was observed (Fig. 2a-e). *L. plantarum* A011, *L. rhamnosus* A012 and *L. rhamnosus* A072 inhibited the growth of all the tested pathotypes of diarrheagenic *E. coli* at 16 h, however, *P. pentosaceus* A074, *L. pseudomesenteroides* A044, *W. cibaria* B3a and *L. pentosus* B1b inhibited the growth of all pathogens at 16 h and 24 h (Fig. 2a-e).

**Fig. 3:**
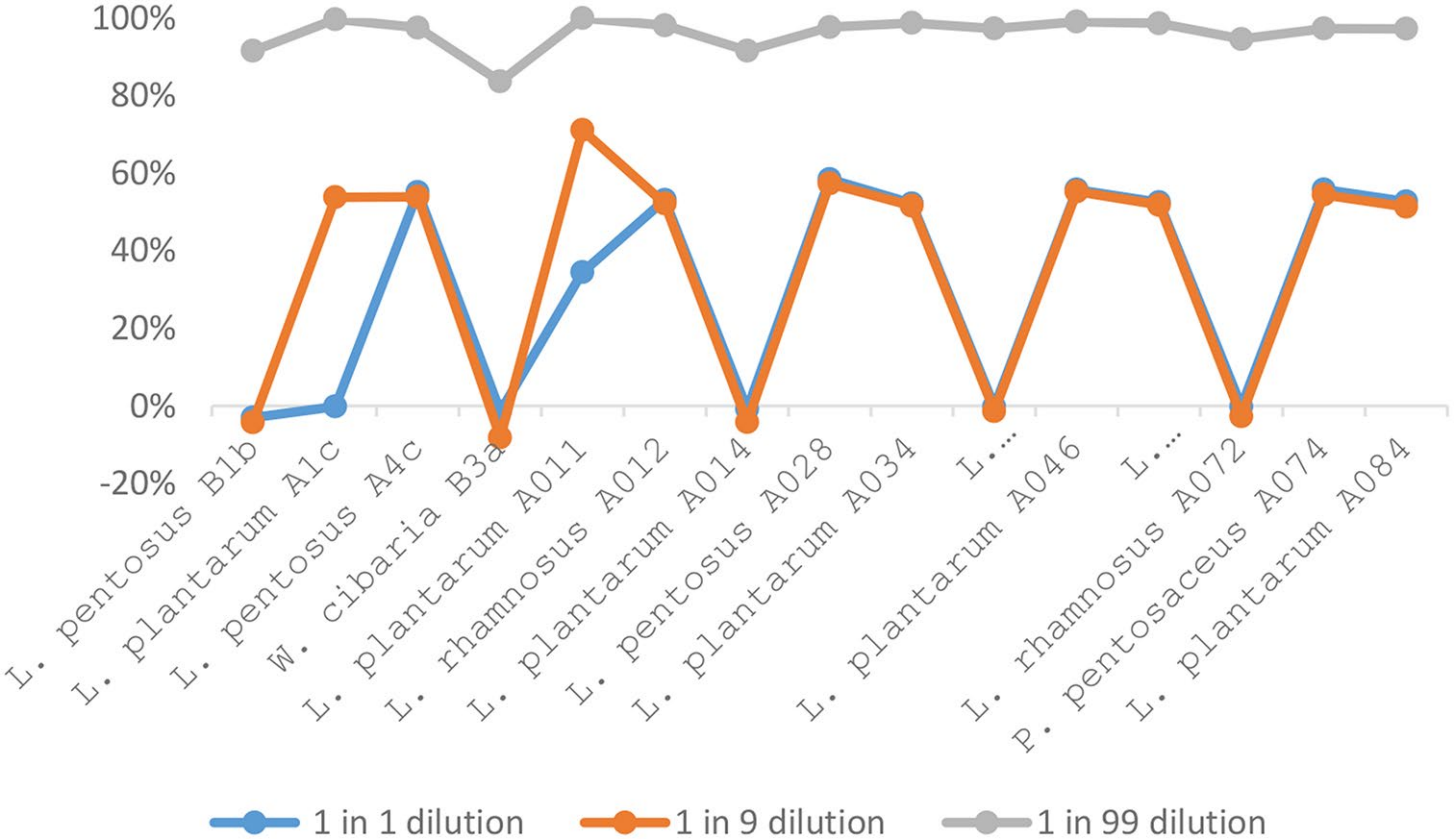
Antibiofilm inhibition of lactic acid bacteria against enteroaggregative *Escherichia coli* strain

Meanwhile, the diluted CFS of the LAB isolates possessed biofilm inhibition at different dilution factor. At 1 in 1 dilution, *L. pentosus* A4c, *L. plantarum* A011, *L. rhamnosus* A012, *L. pentosus* A028, *L. plantarum* A034, *L. plantarum* A046 and *L. plantarum* A084 strains had biofilm inhibition. However, 2 strains of *L. plantarum* (A1c and A011) possessed biofilm inhibition at 1 in 9 dilutions, but 1 in 99 dilutions of all the LAB strains showed biofilm inhibition. Three different dilutions of *L. plantarum* A011 showed appreciable biofilm inhibition (Fig. 3).

**Fig. 4:**
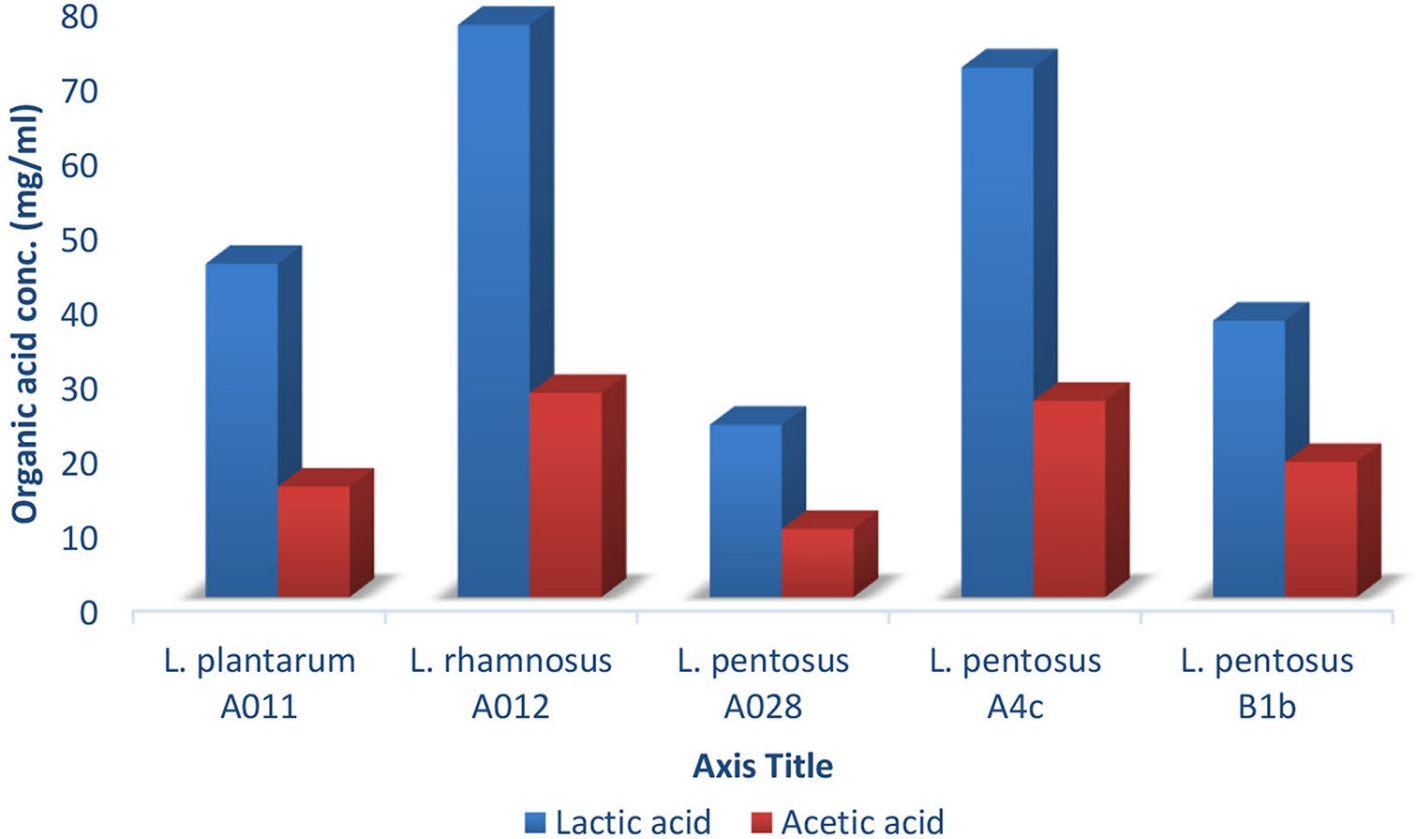
Concentration (mg/mL) of organic acid produced by LAB

The highest organic acids was produced by *L. rhamnosus* A012 (lactic acid 76.81 mgml^− 1^; acetic acid 27.39 mgml^− 1^) while *L. pentosus* A028 produced the least (lactic acid 23.12 mgml^− 1^; acetic acid 9.13 mgml^− 1^) as shown in Fig. 4.

All the tested LAB isolates (100%) were non haemolytic (γ-haemolysis). However, the screened LAB isolates exhibited varying resistance pattern to tested antibiotics. Larger percentage of LAB showed resistance to ampicillin (80%), 26.7% were resistant to tetracycline, while 33.3% showed resistance to erythromycin. However, *L. rhamnosus* A012 was susceptible to all the antibiotics, while *L. plantarum* A011 and *L. pentosus* A4c were resistant to only ampicillin while *L. pentosus* A028 was resistant to only clindamycin (Table 2).

**Table 2:** Antibiotic susceptibility pattern of lactic acid bacteria strains isolated

Lactic acid bacteria strains	Cefoxitin Screen	Ampicillin	Oxacillin	Gentamicin High Level (Synergy)	Streptomycin High Level (synergy)	Gentamicin	Ciprofloxacin	Levofloxacin	Moxifloxacin	Inducible Clindamycin Resistance	Erythromycin	Clindamycin	Linezolid	Daptomycin	Vancomycin	Doxycycline	Tetracycline	Tigecycline	Nutrofurantoin	Rifampicin	Trimethoprim/ Sulfamethoxazole
*L. plantarum* A011	S	R	S	SYN-S	S	S	S	S	S	NEG	S	S	S	S	S	S	S	S	S	S	S
*L. plantarum* A034	S	R	S	S	S	S	S	S	S	NEG	R	R	NI	S	S	S	S	NI	S	S	S
*L. plantarum* A046	S	R	S	S	S	S	S	S	S	NEG	R	S	NI	S	S	S	S	S	S	S	S
*L. plantarum* A084	S	R	S	SYN-S	S	S	S	S	S	NEG	S	S	NI	S	S	S	S	S	S	S	S
*L. plantarum* A1c	S	R	S	S	R	S	S	S	S	NEG	R	S	NI	S	S	S	S	NI	S	S	S
*L. xianqfangensis* B1a2	S	R	S	R	S	S	S	S	S	NEG	S	R	S	S	S	2	S	NI	S	NI	NI
*L. rhamnosus* A012	S	S	S	S	S	S	S	S	S	NEG	S	S	S	S	S	S	S	S	S	S	S
*L. rhamnosus* A072	S	R	S	S	R	S	S	S	S	NEG	R	R	NI	S	S	S	S	S	S	S	S
*L. pentosus* A028	S	S	S	S	S	S	S	S	S	NEG	S	R	S	S	S	S	S	S	S	S	S
*L. pentosus* A4c	S	R	S	SYN-S	S	S	S	S	S	NEG	S	S	S	S	S	S	S	S	S	S	S
*L. pentosus* B1b	S	R	S	SYN-S	S	S	S	S	S	NEG	S	S	S	S	S	S	S	S	S	8	S
*Leuc. pseudomensenteroides* A044	S	R	S	SYN-S	S	S	S	S	S	POS	S	S	NI	S	S	S	R	NI	S	8	S
*Leuc. pseudomensenteroides* A064	S	R	S	SYN-S	S	S	S	S	S	NEG	S	S	NI	S	S	S	R	S	S	S	S
*Ped. pentosaceus* A074	S	S	S	SYN-S	S	S	S	S	S	NEG	S	S	S	S	S	S	R	S	S	S	S
*W. cibaria* B3a	S	8	S	SYN-S	S	S	S	S	S	POS	R	S	S	S	S	S	R	NI	S	S	S

*Note* S represents Susceptible, R represents Resistance, SYN represents Synergy, NEG represents Negative, NI represents Value of susceptibility not indicated

*Note* The breakpoint was adapted from the European Food Safety Authority (EFSA). 2018. 16.3: 5206. Any MIC that is greater than the breakpoint is considered to be resistant. According to EFSA, (2018), the cut off values of lactic acid bacteria to antibiotics are varied, the cut-off values for each LAB specie is therefore used to check their susceptibility or resistance to different antibiotics in the table

### Lactic acid bacterial isolates survived simulated gastrointestinal conditions

Among the 93 LAB isolates screened for their tolerance to low acidic condition, 92.47% of the isolates demonstrated high rate of survival after exposure to pH 3 for 3 h (83. 36–99.84% survival rate) out of which 54.54% of LAB had 1 log_10_ reduction. However, 1.08% LAB isolates had complete growth inhibition (completely killed) while, 6.45% of the isolates produced higher cfu/mL in acidic condition (Supplementary Table 2). Meanwhile, at pH 2, the growth rate was 55.91%, in which 4.3% had 1 log_10_ reduction of cfu/mL, 19.35% had 2 log_10_ reductions, 26.88% had 3 log_10_ reductions and 6.45% had 4 log_10_ reductions after 3 h of incubation. On the other hand, 44.09% isolates had no growth after exposing them to pH 2 for 3 h (Supplementary Table 2).

Furthermore, among the 93 LAB tested for their tolerance to bile, 53.7% reduced in viable cell count but no log reduction, 1 log reduction (31.18%), 2 log reduction (3.22%) while no growth in 0.3% bile (11.83%), however, 17.20% of LAB isolates had increase in viable cell counts. (Supplementary Table 2). *P. pentosaceus* A074, *L. rhamnosus* A072, *L. rhamnosus* A012 and *L. plantarum* A01 had growth in acid and bile environment.

**Fig. 5:**
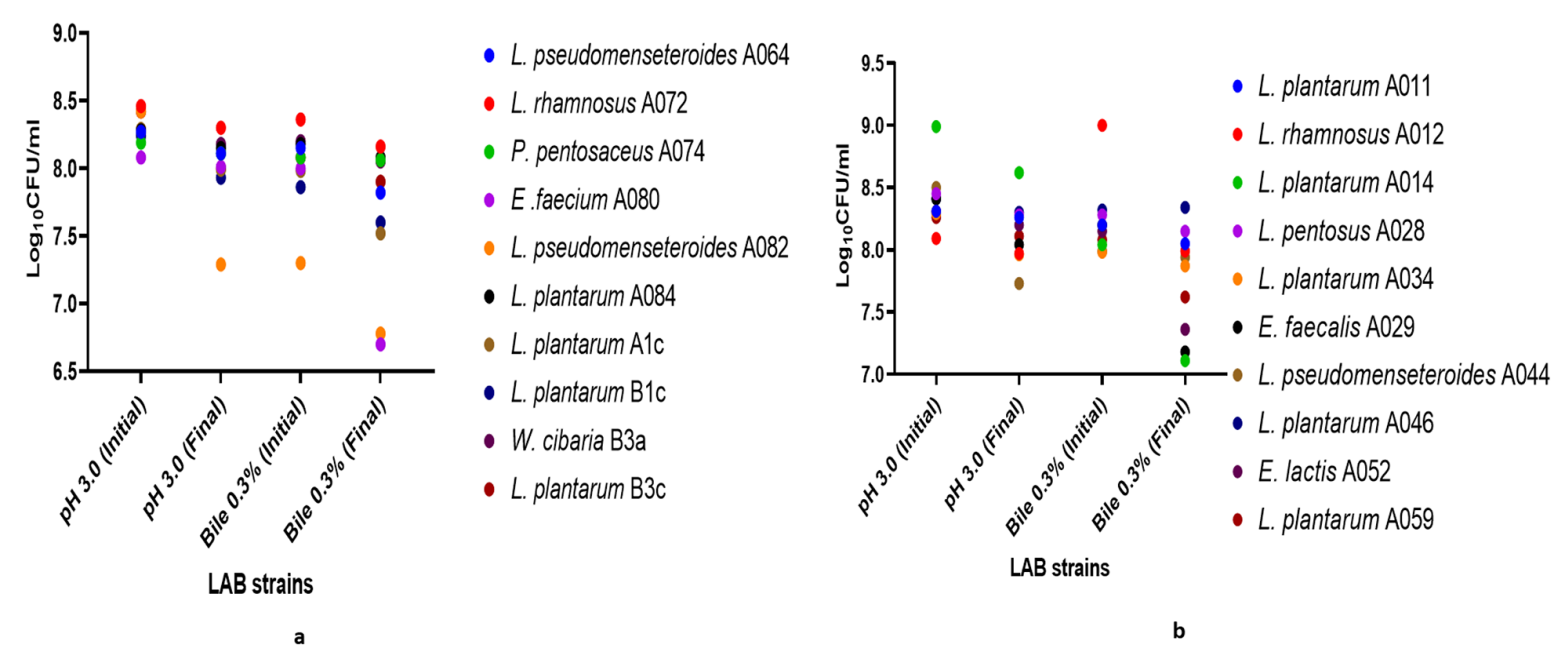
**a** and **b**: Survival of LAB in successive low pH and bile salt supplement

However, successive transit of LAB in acid and bile simulation showed the following strains: *L. plantarum* A011, *L. pentosus* A028, *W. cibaria* B3a, *L. rhamnosus* A072, *P. pentosaceus* A074, *L. plantarum* A046 and *L. pseudomesenteroides* A084 had no log reduction but reduced in cell counts (Fig. 5a-b). In the same manner, the viable cell counts of *L. rhamnosus* A012, *L. rhamnosus* A072, *L. pentosus* A4c, *L. pseudomesenteroides* A044, *L. plantarum* A1c and *L. pseudomesenteroides* A064 isolates reduced with1 log as shown in Fig. 5a-b).

The adhesive and affinity potential evaluated through cell surface hydrophobicity using *n*-hexadecane and xylene showed that LAB isolates exhibited high affinity rates which ranged between 0.29% and 90.73% (*n*-hexadecane); 0.36% and 71.88% (xylene). The hydrophobicity of LAB strains to *n*-hexadecane was 67.74% while hydrophobicity of the strains to xylene was 56. 99%. As shown in the results, *L. pseudomesenteroides* A030 had highest hydrophobicity with 90.57% (*n*-hexadecane) and 75.80% (xylene). Meanwhile, the hydrophobicity of *L. rhamnosus* A012 was 25.01% (*n*-hexadecane); 15.40% (xylene), *L. rhamnosus* A072 with 11.63% (*n*-hexadecane); 8.01% (xylene) and *L. plantarum* A011 was 9.27% (*n*-hexadecane); 14.41% (xylene) (Supplementary Table 3).

The auto-aggregation result for selected LAB strains showed that each strain had the ability to auto-aggregate, and among these strains *L. rhamnosus* A072 showed the highest autoaggregation percentage of 46.37 ± 0.01% (Table 3) followed by *L. pentosus* A028 (38.20%), *L. xiangfangensis* B1a2 had 37.06%, *L. rhamnosus* A012 (32.52%), *W. cibaria* B3a (32.68 ± 0.42%) and *L. plantarum* A011 (20.83 ± 0.27%).

**Table 3:** Autoaggregation of LAB and co-aggregation of LAB with diarrhoeagenic *E. Coli* strains

Strains	Autoaggregation % of LAB	Coaggregation % of LAB with EPEC (H62E)	Coaggregation % of LAB with EAEC (H40C)	Coaggregation % of LAB with EIEC (H68D)	Coaggregation % of LAB with ETEC (H40B)	Coaggregation % of LAB with STEC (H77B)
*L. plantarum* A011	20.83 ± 0.27	19.17 ± 0.03	-17.78 ± 0.17	-13.59 ± 0.00	-90.08 ± 0.02	2.19 ± 0.00
*L. rhamnosus* A012	32.52 ± 0.34	33.34 ± 0.17	-21.63 ± 0.02	0.95 ± 0.02	-82.60 ± 0.01	2.17 ± 0.00
*L. pentosus* A028	38.20 ± 0.42	18.29 ± 0.01	-42.03 ± 0.02	-36.58 ± 0.04	-19.80 ± 0.01	-2.43 ± 0.01
*L. plantarum* A034	-26.36 ± 0.17	NA	NA	NA	NA	NA
*Leuc. pseudomensenteroides* A044	20.89 ± 0.00	-26.93 ± 1.59	-46.19 ± 0.01	-17.18 ± 0.03	-70.63 ± 0.01	-9.95 ± 0.01
*L. plantarum* A046	11.51 ± 0.13	11.09 ± 0.03	-23.10 ± 0.09	6.64 ± 0.01	-15.65 ± 0.00	-0.88 ± 0.01
*Leuc. pseudomensenteroides* A064	15.02 ± 0.01	NA	NA	NA	NA	NA
*L. rhamnosus* A072	46.37 ± 0.01	NA	NA	NA	NA	NA
*Ped. pentosaceus* A074	20.32 ± 0.00	30.22 ± 0.05	-15.15 ± 0.01	6.52 ± 0.02	-13.69 ± 0.03	-1.11 ± 0.01
*L. plantarum* A084	9.25 ± 0.36	-0.16 ± 0–01	-0.62 ± 0.01	4.37 ± 0.01	-29.11 ± 0.03	-41.72 ± 0.01
*L. plantarum* A1c	25.77 ± 0.01	-25.88 ± 0.30	-32.82 ± 0.04	-1.48 ± 0.00	-40.14 ± 0.00	-34.91 ± 0.00
*L. pentosus* A4c	24.28 ± 0.01	26.68 ± 0.03	-60.42 ± 0.09	-19.34 ± 0.04	-7.80 ± 0.00	5.63 ± 0.00
*L. xianqfangensis* B1a2	37.06 ± 0.23	NA	NA	NA	NA	NA
*L. pentosus* B1b	20.23 ± 0.41	24.98 ± 0–03	-37.81 ± 0.04	16.99 ± 0.01	-101.82 ± 1.59	-13.91 ± 0.01
*W.cibaria* B3a	32.68 ± 0.04	-28.70 ± 0.33	-46.07 ± 0.05	-7.75 ± 0.06	-32.13 ± 0.02	0.00 ± 0.01

*Note* Mean ± Standard deviation. The negative value in co-aggregation results indicates the LAB strains could not form co-aggregation with the *pathogenic E.coli* strains. NA indicates the LAB strains that were not inolved in co-aggregation assay due to their resistance to antibiotics

The co-aggregation of selected LAB strains with diarrheagenic *E. coli* strains showed that LAB strains possess the abilities for aggregation with diarrheagenic *E. coli*. *L. rhamnosus* A012 exhibited highest coaggregation percentage of 33.34 ± 0.17% with EPEC H62E, followed by *P. pentosaceus* A074 with 30.2% (Table 3). In addition, the coaggregation percentage of *L. plantarum* A011 with EPEC H62E was 19.17 ± 0.03%, *L. pentosus* A028 was 18.29 ± 0.01%, *L. plantarum* A046 (11.09 ± 0.03%), *L. pentosus* A4c (26.68 ± 0.03%) and *L. pentosus* B1b was 24.98 ± 0.03%. *L. pseudomesenteroides* A044, *L. plantarum* A1c, *L. plantarum* A084 and *W. cibaria* B3a could not co-aggregate with EPEC (H40C). No LAB strains could co-aggregate with EAEC H40C, however, it was observed that *L. rhamnosus* A012 was able to competitively co-aggregate with EIEC H68D, EPEC H62E and STEC H77B (Table 3).

### Immunomodulatory activity of lactic acid bacteria

General observation following cyclophosphamide intraperitoneal injection.

**Fig. 6:**
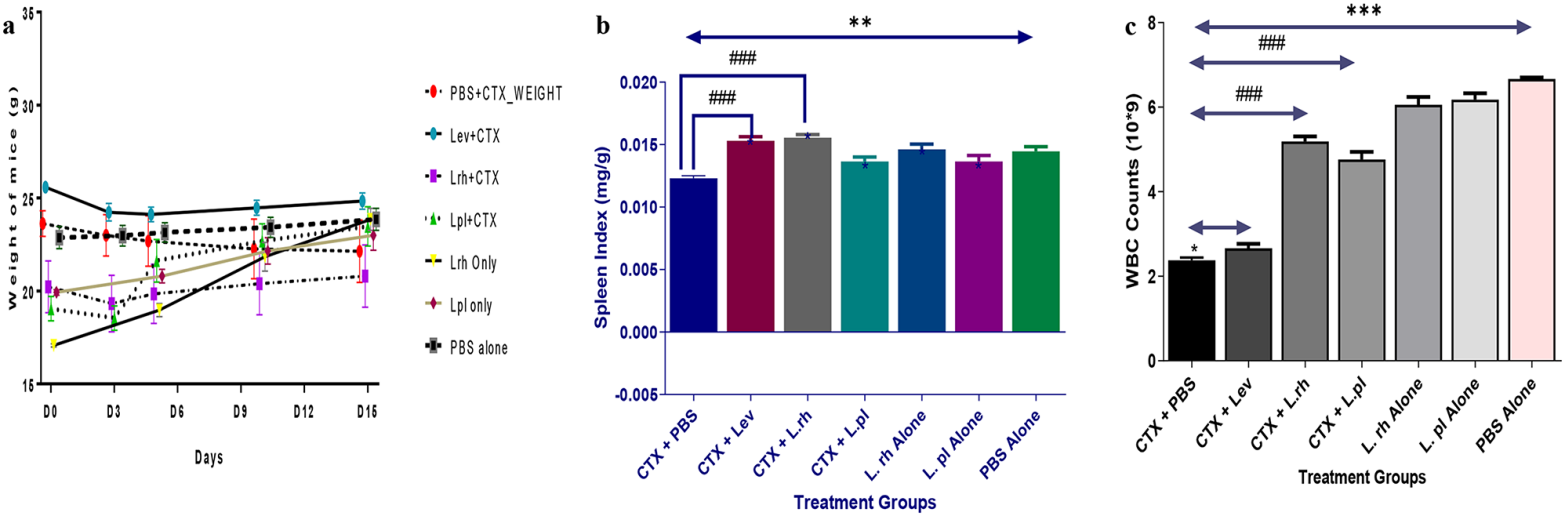
**a-c**: The ameliorating effect of probiotic on weight, spleen index and white blood cells count of immunosuppressed mice. *Note* PBS alone (Phosphate buffer saline alone – immunocompetent), CTX+PBS (cyclophosphamide and PBS – immunocompromised), CTX+Lev (cyclophosphamide and levamisole hydrochloride - positive control), CTX+L.rh (cyclophosphamide and *L. rhamnosus* A012), CTX+L.pl (cyclophosphamide and *L. plantarum* A011), L.rh alone (*L. rhamnosus* A012- immunocompetent) and L.pl alone (*L. plantarum* A011- immunocompetent. **a** - *P< 0.05 CTX+PBS vs CTX+Lev or CTX+L.rh or CTX+L.pl and PBS alone showed a normal body weight gain. **b** - **P<0.01 PBS alone vs CTX+PBS, ^###^ P<0.001 CTX+PBS vs CTX+Lev or CTX+L.rh or CTX+L.pl. **c**- ***P< 0.001 PBS alone vs CTX+PBS, ^#^P < 0.05 CTX+PBS vs CTX+Lev, ^###^P< 0.001, significant difference between CTX+PBS and CTX+L.rh or L.rh alone (One-way ANOVA followed by Turkey’s multiple comparisons post-hoc test)

The mice in group 1, 2, 3 and 4 showed lassitude, lackluster, fur piloerection, and lack of enthusiasm to environmental activity on day 1 after the intraperitoneal administration of CTX, they had loose-watery bowel movements, with reduction in their food and water intake especially on day 2–3 after the intraperitoneal administration of CTX. There was no weight loss at the onset of the experiment (Fig. 6a). However, following administration of cyclophosphamide (i.p), the immunosuppressed groups (CTX + PBS, CTX + Lev, CTX + Lev and *L. rh* + CTX) experienced a drastic weight loss when compared with immunocompetent groups (L .rh alone L.pl alone and PBS alone) as shown in Fig. 6a. Nonetheless, after the intervention with lactobacilli and levamisole hydrochloride, there was a significant increase (*p*˂0.05) in body weight of mice in these treatment groups compared to mice in the untreated group (CTX + PBS group). This finding infers the ability of *Lactobacillus* sp in restoring the weight loss due to administration of cyclophosphamide. Groups 5, 6 and 7 only had gradual body weight increase (Fig. 6a).

The spleen index value in CTX + PBS group was significantly reduced compared to CTX + Lev, *L. rh* + CTX and PBS alone groups with *p*˂0.0001. However, a reduction was also observed in *L. plant* + CTX group compared to CTX + Lev, *L. rh* + CTX and PBS groups but with no significance difference (Fig. 6b). There was also a significant reduction in immunocompetent groups (*L. rh* alone and *L. plant* alone) as compared to CTX group with p value ˂0.001 (Fig. 6b). The white blood cell count of the experimental mice was increased significantly in CTX + Lev, *L. rh* + CTX compared to CTX + PBS and PBS groups with significant difference of *p* < 0.0001 (Fig. 6c).

**Fig. 7:**
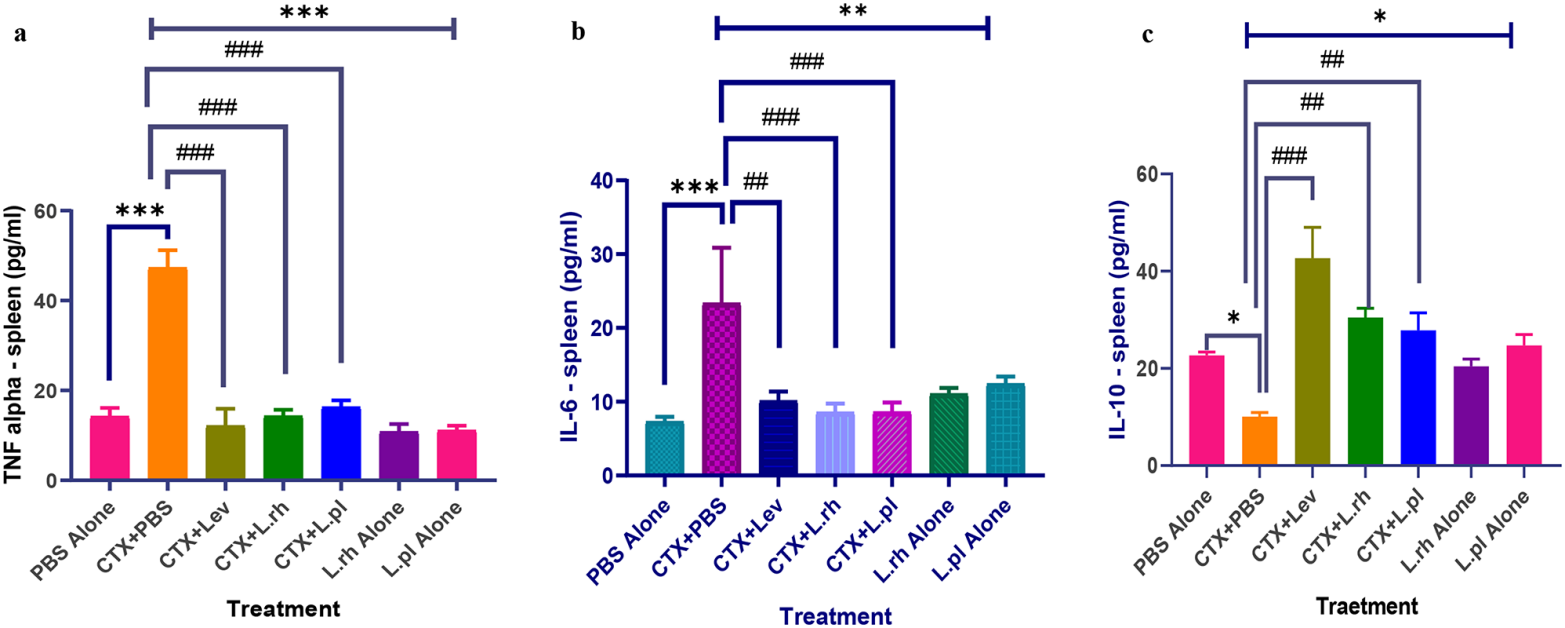
**a-c**: Ameliorating effect of *L. rhamnosus* A012 and *L. plantarum* A011 on TNF-α, IL-6 and IL-10 concentrations in the spleen of immunosuppressed mice. Each column indicates mean ± SEM (n = 4). PBS alone (Phosphate buffer saline alone – immunocompetent), CTX+PBS (cyclophosphamide and PBS – immunocompromised), CTX+Lev (cyclophosphamide and levamisole hydrochloride - positive control), CTX+L.rh (cyclophosphamide and *L. rhamnosus* A012), CTX+L.pl (cyclophosphamideand *L. plantarum* A011), L.rh alone (*L. rhamnosus* A012- immunocompetent) and L.pl alone (*L. plantarum* A011- immunocompetent. **a**: ***P< 0.001 PBS alone vs CTX+PBS, ^###^p< 0.001 CTX+PBS vs CTX+Lev or CTX+L.rh or CTX+L.pl. **b**: ***P< 0.001 PBS alone vs CTX+PBS, ^##^p< 0.01 CTX+PBS vs CTX+Lev and ^###^p< 0.001 significant difference between CTX+PBS vs CTX+L.rh, or CTX+L.pl. Figure 7c: *P< 0.05 PBS alone vs CTX+PBS, ^##^p< 0.01 and ^###^p< 0.001 significant difference between CTX+PBS vs CTX+L.rh, or CTX+L.pl or CTX+Lev. (Analysis is One-way ANOVA followed by Turkey’s multiple comparisons post-hoc test)

**Fig. 8:**
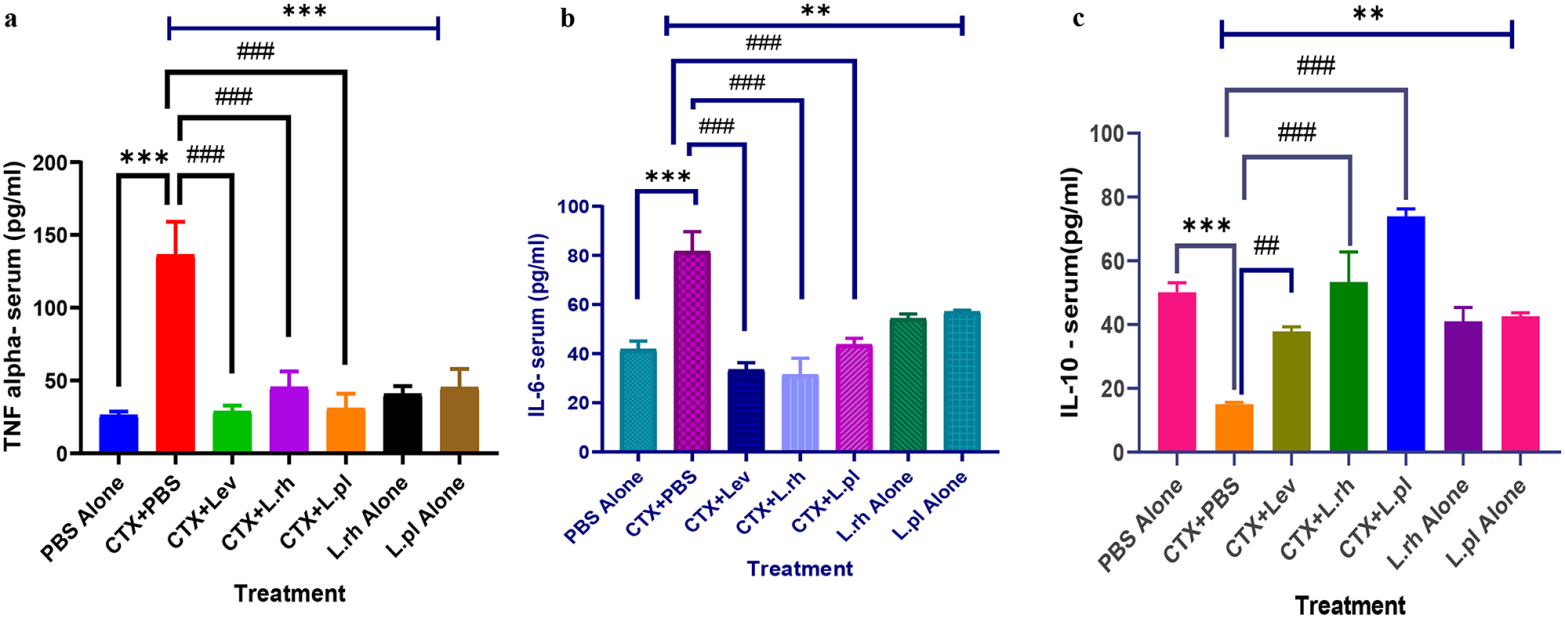
Effect of *L. rhamnosus* A012 and *L. plantarum* A011 on TNF-α, IL-6 and IL-10 concentrations in the serum of immunosuppressed mice. Each column indicates mean ± SEM (n = 4). PBS alone (Phosphate buffer saline alone – immunocompetent), CTX+PBS (cyclophosphamide and PBS – immunocompromised), CTX+Lev (cyclophosphamide and levamisole hydrochloride - positive control), CTX+L.rh (cyclophosphamide and *L. rhamnosus* A012), CTX+L.pl (cyclophosphamideand *L. plantarum* A011), L.rh alone (*L. rhamnosus* A012- immunocompetent) and L.pl alone (*L. plantarum* A011- immunocompetent). **a** & **b**: ***P< 0.001 significance difference between PBS alone and CTX+PBS, while ^###^p< 0.001 is the significant difference between CTX+PBS and CTX+L.rh, or CTX+L.pl or CTX+Lev. **c**: ***P< 0.001 PBS alone vs CTX+PBS, ^##^p< 0.01 CTX+PBS vs CTX+Lev and ^###^p< 0.001 significant difference between CTX+PBS vs CTX+L.rh, or CTX+L.pl. (Analysis-One-way ANOVA followed by Turkey’s multiple comparisons post-hoc test)

The effect of treatment with *L. rhamnosus* A012 and *L. plantarum* A011 on both serum and spleen level of TNF-α, IL-6 and IL-10 in mice is presented in Fig. 7a and c, & Fig. 8a and c. Administration of CTX in mice caused significant increase in TNF-α level (*p* < 0.001), however, *L. rhamnosus* A012 and *L. plantarum* A011 treatment significantly decreased (*p* < 0.001) the spleen and serum level of TNF-α showing ameliorative effect of *Lactobacillus* sp. (Figures 7a and 8a). In like manner, intervention treatment with *L. rhamnosus* A012 and *L. plantarum* A011 also significantly reduced the level of IL-6 in both serum and spleen of the mice as shown in Figs. 7b and 8b.

However, the administration of CTX significantly decreased *(p* < 0.05) the serum and spleen level of IL-10, but this was averted by the intervention of *L. rhamnosus* A012 and *L. plantarum* A011 (Figs. 7c and 8c). Administration of *Lactobacillus* species significantly (*p <* 0.001) reduced the concentrations of both TNF-α and IL-6 in the serum and tissue of mice treated with *L. rhamnosus* A012 and *L. plantarum* A011 in comparison with the CTX + PBS treated mice, ameliorating the effect of cyclophosphamide in the treated groups (Fig. 7a and b). In addition, there was a significant reduction of IL-10 in the serum and spleen of animals in CTX + PBS group compared to PBS group (control). However, after treating the animals with *L. rhamnosus* A012 and *L. plantarum* A011, the quantity of IL-10 produced in the serum and spleen of animals in these groups significantly increased (*p* < 0.01) showing anti-inflammatory ability of *L. rhamnosus* A012 and *L. plantarum* A011 and the ameliorating effect of LAB on cyclophosphamide induced immunosuppression mice.

## Discussion

Breast milk was thought to be sterile until new facts emerge about the presence of beneficial bacteria, which protects infants from gastroenteritis infections, asthma and allergy [46]. Likewise, the presence of LAB in the GIT determines a healthy gut and can protect a child from infection [47]. In the current study, the diversity of LAB cultured from human breast milk and child faeces shows 93 lactic acid bacteria of 15 different species belonging to 4 genera viz.: *Lactobacillus*, *Enterococcus*, *Weisella*, *Leuconostoc*, and *Pediococcus.* We observed that *Lactobacillus* was the most frequent genera identified and *L. plantarum* was the most abundant specie in human breast milk (36.36%) and neonates’ faeces (18.42%), respectively. This collaborates other studies in which *Lactobacillus* species were abundantly identified from breast milk [48–50] and faeces of breastfed infants [51]. This is however contrary to a report where *S. epidermidis* and *E. faecalis* were predominantly identified from breast milk and neonates’ faeces [33]. Lactic acid bacteria have also been isolated from cattle, pigs, pickles, and sausage [37, 52], showing that LAB can also be found in other sources. In this study, *L. xiangfangensis* was isolated from breast milk; this is novel specie that was previously isolated from Chinese pickle [53]. In addition, *E. thailadicus* was identified from breast milk, supporting the claim that human milk-faecal microbiome consists of thousands of species of microorganisms that are yet to be identified [40]. *L. plantarum*, *L. rhamnosus* and some other strains were found in both human breast milk and neonate’s faeces, this supports the claim that composition of LAB in faeces of exclusive breastfeeding neonates usually reflect mothers breast milk [54].

An essential criterion for the selection of probiotic strains is the ability of LAB strains to exert antimicrobial effect against pathogenic microorganisms, through the production of some organic acid metabolites such as lactic, acetic, butyric, propionic acid. Hence, this study reports that *L. rhamnosus* A012, *P. pentosaceus* A074, *L*. *plantarum* A023, *L. pseudomesenteroides* A044, *L. pentosus* B1b, *L. pentosus* A028 *L. rhamnosus* A072, *L. plantarum* A011, *L. pentosus* A4c, *L. plantarum* A051, *L. plantarum* A051 and *L. plantarum* A014 produce significant inhibition against all diarrheagenic *E. coli* strains tested through various methods such as agar well diffusion, agar overlay, and time-kinetic study. Similarly, antimicrobial activity of LAB isolated from breast milk and neonates’ faeces against *E. coli*, *Shigella* spp., *Pseudomonas* spp., *Salmonella* spp., and different pathotypes of diarrheagenic *E. coli* strains have been reported [51, 55]. In like manner, LAB isolates from different countries were also found to produce antimicrobial activity against pathogenic fungi such as *Candida albicans* and Gram-positive bacteria like *Bacillus cereus*, *Staphylococcus albus*, *Streptococcus faecalis*, *Streptococcus thermophilus*. Similarly, LAB from fermented food produce antimicrobial activity against diarrheagenic *E. coli* strains [31]. Furthermore, co-culturing LAB strains with diarrheagenic *E. coli* strains also showed its probiotic potential by completely inactivating the organisms within 24 h of contact. Interestingly, *L. rhamnosus* A072, *L. plantarum* A011, *L*. *pentosus* A028, *L. plantarum* A084, *L. rhamnosus* A012, *L. pentosus* B1b, and *P. pentosaceus* A074 inhibited all the diarrheagenic *E. coli* strains between 8 h and 16 h of contact. This study correlates the report of [29, 33, 36, 37] and [30].

The lactic acid bacteria strains in this study exert their antimicrobial activity against the pathogenic strains by releasing inhibitory substances such as lactic acid, acetic acid, and other compounds against diarrheagenic *E. coli* strains. This claim is supported by high quantity of lactic and acetic acid released by some LAB strains, such as *L. rhamnosus* A012, thereby corroborating the report of [37] and [56].

The production of biofilm by many pathogenic strains such as *S. aureus*, *S. viridans*, *K. pneumonia*, *P. mirabilis*, *P. aeruginosa* and *E. coli* enhances antimicrobial resistance. Our study shows that lower dilution of CFS possessed antibacterial activity against EAEC strain 042, while at the high dilution of 1:100, *L. plantarum* A011 produced outstanding anti-biofilm potential showing the interference of LAB with the biofilm integrity of EAEC strain 042 to completely prevent biofilm formation. The antibiofilm mechanisms could be their ability to produce inhibitory substance or ability to form auto- and co-aggregation. Hence, the antibiofilm activity exhibited by *L. plantarum* A011 and other LAB validates the reports that CFS of *Lactobacillus* strains could produce antibiofilm activities against pathogens [57]; [58, 59].

The survival of LAB in gastrointestinal condition is an essential feature that must be possessed by potential oral probiotics for the strains to survive the gastrointestinal effect and get to the colon. In line with this, the current study reports the survival of LAB strains in both pH 2.0 and 3.0, although smaller percentage of the LAB strains survived pH 2.0, larger percentage of the strains survived pH 3.0 with the viable cells range of 8.05 and 8.16 log. However, few LAB strains were able to grow in the bile salt as their survival rate was over 100%. At the initial exposure to 0.3% bile salt, their viable cells reduced but were found improved and survived after 4 h of exposure to the stress condition indicating that these LAB strains may contain certain functional proteins that protect and allow better survival to the bile stress condition [60]; [61]; [62]; [63]. The identified potential probiotics strains through survival in gastrointestinal conditions in this study include *L. plantarum* A011, *P. pentosaceus* A074, *L. rhamnosus* A072, *L. rhamnosus* A012 and *L. pentosus* A4c among others, this is in line with the characteristics of the potential probiotics that thrive in gastrointestinal conditions [64]; [65]; [66]. In addition, these LAB isolates survived the adverse environment of consecutive gastrointestinal transit with survived viable cells range of 6.78 to 8.42 log. The survival ability of LAB isolates through GI tract condition may be due to the production of organic acid and/or production of resistant cell wall material [67], the production of bile resistance gene (bsh-1 and bsh-2) in LAB cells [68] or their evolutionary stress-sensing system defense mechanism which allow them to migrate across the intestine [69, 70], the following strains *P. pentosaceus* A074, *L. rhamnosus* A012, *L. pentosus* A4c, *L. plantarum* A011 and *L. rhamnosus* A072 maintain their viability and falls within the range of 1.0 × 10^6^ and 1.0 × 10^10^ in simulated gastrointestinal environment [71].

Another essential criterion in the selection of probiotic strain is its ability to adhere to mucus and/or human epithelial cells. The adherence of LAB strains to mucus layer makes use of specific and non-specific mechanisms to exclude the pathogenic organism [72, 73]. However, according to van Zyl et al. [74] the activities of LAB are strain specific, therefore, the mechanisms of action used by one bacterial species to exclude other bacteria from the intestinal tract differ and may comprise microbe-microbe interactions mediated by binding to the mucosal interface of the host at specific sites of attachment [74]. Cell surface hydrophobicity, a biophysical measurement uses cell-surface interaction mechanism. Therefore, LAB strains with high cell surface hydrophobicity showed affinity to non-polar interface with high adhesion to cell-surface interphase, thereby use non-specific mechanism to exclude the pathogens and prevent them from colonizing mucus cell lines. The current study demonstrated that the screened LAB isolates have the potential to colonize and adhere to epithelial layer of the intestine with the isolates displaying both high and low affinity to *n-*Hexadecane and Xylene, thereby, producing a non-specific adherence through cell surface hydrophobicity. It has been reported that LAB isolates from faeces possess higher affinity to surface hydrophobicity [75], this is in accordance with the current study as LAB isolates from fecal samples demonstrated high percentage of cell surface hydrophobicity more than the isolates from breast milk. We therefore report LAB isolates to possess a protective ability on the host epithelia layer supporting [50] that *Lactobacillus* and *Bifidobacterium* species protect the epithelia layer of intestine from mechanical damage and bacterial infection.

The LAB strains in this study demonstrated both auto-aggregation and co-aggregation quality as it was observed in L. *rhamnosus* A012, *L. rhamnosus* A072, *L. pentosus* B1b and *P. pentosaceus* A074. This shows that the LAB cell form biofilm which protect them from environmental stress or any irrational from response host cells [57–59]. LAB also form aggregate around pathogens of the same species or different species, these are destroyed by their production of organic acid [75]. The selected LAB strains excluded the diarrheagenic *E. coli* in the co-aggregation assay, and competitively displayed EPEC and EIEC, this indicates the ability of LAB to protect the GIT corroborating earlier researchers’ reports that LAB possess the ability to produce adhesion property in-vitro forming a protective barrier through autoaggregation and co-aggregation [75–77].

According to WHO guidelines on the use of probiotics, probiotic strain should not possess any antibiotic resistance gene that is transferrable as this can pose a potential risk on human health [648]. Consistent with early research [72, 78], this study found that nearly all screened LAB isolates were susceptible to gentamicin, vancomycin, and kanamycin, in addition, the LAB strains were susceptible to tetracycline except *P. pentosaceus* A074, *L. pseudomesenteroides* A064, and *W. cibaria* B3a. The high resistance of LAB strains to ampicillin support [79]. The LAB strains were also susceptible to erythromycin and clindamycin [72, 80] contrary to other report [81] and [82]. It is noteworthy that *L. rhamnosus* A012 was susceptible to all the 21 antibiotics used, correlating [83] report on *L. rhamnosus* GG being safe. However, *L. rhamnosus* A072, was resistant to clindamycin, ampicillin, streptomycin and erythromycin establishing the notion of strain specificity of probiotic. Therefore, we report that *L. rhamnosus* A012 could be a potential probiotic but cannot be co-administered with these antibiotics. However, *L. pentosus* B1b, *L. pentosus* A4c, and *L. plantarum* A011 strains could be co-administered with antibiotics as there has not been a report of their gene carry resistance gene. Potential probiotics must not haemolyse blood [84]. This requirement was followed in this study as all the LAB strains possess γ-haemolysis (non-haemolytic) [85, 86].

Ability of probiotic strain to modulate immune responses is a desirable criterion of probiotics. Bifidobacteria and lactic acid bacteria play essential role using defense mechanism to protect host cell, they stimulate, modulate, and regulate immune system [87, 88]. Cyclophosphamide, as a chemotherapy in cancer treatment has also been clinically confirmed to be used in immune suppressor for nephrotic syndrome, granulomatosis with polyangiitis, and organ transplant among other conditions [89–91], hence, the reason for its selection in modelling immunosuppression. In this study, the administration of CTX resulted in weight loss, loss of appetite, inaction, uncontrollable turning of neck and diarrhea in immunosuppressed mice correlating the report of [91] where administration of CTX resulted in immunological damage. However, the treatment with levamisole hydrochloride and *Lactobacillus* species alleviated the immunological damage previously observed by significantly accelerating the weight increase, gastrointestinal stability and recovering of white blood cells in the treated mice, this indicates protective features of *Lactobacillus* strains [87]. The spleens accommodate the immune cells, which protect the host and play a major role in regulating the immune responses [92, 93]. From our report, *Lactobacillus* alleviated the spleen injury caused by CTX to the immune organ thereby, protecting the spleen from further damage, correlating the previously reported work [89, 90].

TNF-α triggers the immune system molecules to induce neutrophil activation [88]. In this study, administration of CTX triggered immunosuppression thereby elevating the pro-inflammatory cytokines in mice. However, oral administration of *Lactobacillus* species reversed the effect and prevented the excessive rise in the concentration of TNF-α and IL-6. Excessive production of pro-inflammatory cytokines can also result in apoptosis of epithelial cell, however, the intervention of *Lactobacillus* sp has been reported to ameliorate this effect as it down-regulated the production of TNF-α and IL-6 in the spleen, this has also been reported by [94] and [88], where *Lactobacillus* strains decreased the expression of TNF-α in contrast to [89] report in which *Lactobacillus* sp upregulated TNF-α. Meanwhile, healthy, and immunocompetent mice showed normal expression of TNF-α supporting the claim that TNF-α might be very low in a healthy individual [94].

Interleukin-6 response triggers the pro-inflammatory and an anti-inflammatory profile [95]. The data presented in this study showed that IL-6 was upregulated with administration of CTX but downregulated with the intervention of *Lactobacillus* species and levamisole hydrochloride (*p* < 0.001). This supports the report of [94] and [89], in which *Lactobacillus* strains modulate the production of IL-6 cytokines.

IL-10, an anti-inflammatory cytokine, regulates the production of pro-inflammatory cytokines to maintains the immune system balance. IL-10 binds to IL-10R1 to initiate the signal transduction activity using Jak-STAT pathway [88]. Through this, intestinal epithelial barrier is strengthened and serve as the central regulator of the inflammatory response [88, 95]. In the current study, the level of IL-10 was significantly elevated upon *Lactobacillus* treatment. Treatment with *Lactobacillus* species upregulated the production of IL-10, thereby strengthening and protecting the cells from injuries. In this study, *L. plantarum* A011 and *L. rhamnosus* A012 possessed anti-inflammatory effect, as these strains elevated the level of IL-10 in both serum and tissue than the standard drug (Levamisole hydrochloride). This indicates the ameliorating effect of Lactobacillus strains to restore gut microbes and damaged intestinal epithelial barrier. Our results, therefore, agrees with [89, 90, 96] and [88] claim, that *Lactobacillus* strains modulate IL- 10 response in immunosuppressed mice. Thus, *L. plantarum* A011 and *L. rhamnosus* A012 have good potential to maintain the intestinal immune balanced through stimulation and regulation of cytokines (TNF-α, IL-6 and IL-10).

This study demonstrated the anti-diarrheagenic *E. coli* activities of lactic acid bacteria isolated from human breast milk and infant’s faeces in vitro and their potentials to function as probiotic candidate in ameliorating the damaged effect of immune deficiency. The two selected *Lactobacillus* strains, *L. plantarum* A011 and *L. rhamnosus* A012 demonstrates promising probiotic properties both in *vitro* and *in vivo.*

## Electronic supplementary material

Below is the link to the electronic supplementary material.

Supplementary Material 1

## Data Availability

The 16 S sequences were deposited in European Molecular Biology Laboratory (EMBL) with accession number PRJNA628165 (https://www.ncbi.nlm.nih.gov/sra/PRJNA628165).

## References

[1] Dawit D, Kumalo E, Yasin Y, Hala Y. Assessment of knowledge attitude & practice of child care givers towards oral rehydration treatment in under 5 children in Wolaita Sodo town. J Biology Agric Healthc. 2016;7(4):3–10.

[2] World Health Organization (WHO). 2017. Diarrhoeal disease - World Health Organization (WHO). https://www.who.int/news-room/fact-sheets/detail/diarrhoeal-disease

[3] UNICEF. 2019. Over 300,000 children under five died from diarrhoeal diseases … UNICEF. https://www.unicef.org/turkiye/en/node/2296

[4] UNICEF. 2021. UNICEF Data: Monitoring the situation of children and women Diarrhoea. https://data.unicef.org/topic/child-health/diarrhoeal-disease/ updated January 2024.

[5] Global Burden of Diarrhoeal Diseases Collaborators. 2015. Estimates of global, regional, national morbidity, mortality, and aetiologies of diarrhoeal diseases: a systematic analysis for the Global Burden of Disease Study 2015. Lancet Infect Dis 2017; 17: 909–948. 10.1016/S1473-3099(17)30276-1 PMC5589208 28579426

[6] Mokomane M, Kasvosve I, de Melo E, Pernica JM, Goldfarb DM. The global problem of childhood diarrhoeal diseases: emerging strategies in prevention and management. Therapeutic Adv Infect Disease. 2018;5(1):29–43. 10.1177/2049936117744429 PMC5761924 29344358

[7] UNICEF. 2016. At a glance: Nigeria. http://www.unicef.org/infobycountry/nigeria_statistics.html. Accessed September.

[8] Pakbin B, Brück WM, Rossen JWA. Virulence factors of enteric pathogenic Escherichia coli: a review. Int J Mol Sci. 2021;22(18):9922. 10.3390/ijms22189922. PMID: 34576083; PMCID: PMC8468683. 34576083 10.3390/ijms22189922 PMC8468683

[9] Bolarinwa OA, Tadesse Tessema Z, Frimpong JB, Seidu A-A, Opoku Ahinkorah B. Multi-level analysis and spatial interpolation of distributions and predictors of Childhood Diarrhea in Nigeria. Environ Health Insights. 2021;15. 10.1177/11786302211045286. 10.1177/11786302211045286 PMC8529312 34690501

[10] Platts-Mills JA, Babji S, Bodhidatta L, Gratz J, Haque R, Havt A, McCormick BJ, McGrath M, Olortegui MP, Samie A, Shakoor S, Mondal D, Lima IF, Hariraju D, Rayamajhi BB, Qureshi S, Kabir F, Yori PP, Mufamadi B, Amour C, Carreon JD, Richard SA, Lang D, Bessong P, Mduma E, Ahmed T, Lima AA, Mason CJ, Zaidi AK, Bhutta ZA, Kosek M, Guerrant RL, Gottlieb M, Miller M, Kang G, Houpt ER. Pathogen-specific burdens of community diarrhoea in developing countries: a multisite birth cohort study (MAL-ED). Lancet Global Health. 2015;3:9. 10.1016/S2214-109X(15)00151-5 PMC7328884 26202075

[11] Jain A, Shah D, Das S, Saha R, Gupta P. Aetiology and outcome of acute diarrhoea in children with severe acute malnutrition: a comparative study. Public Health Nutr Epub 2019 Nov. 2020;8:1–6. 10.1017/S1368980019003069 PMC10200492 31699164

[12] Okeke IN. Diarrhoegenic *Escherichia coli* in sub-saharan Africa: status, uncertainties and necessities. J Infections Developing Ctries. 2009;3:11, 817. 10.3855/jidc.586 20061678

[13] Seidman JC, Johnson LB, Levens J, Mkocha H, Muñoz B, Silbergeld EK, West SK, Coles CL. 2016. Longitudinal comparison of antibiotic resistance in diarrhoeagenic and non-pathogenic *Escherichia coli* from young Tanzanian children. *Frontiers in Microbiology*, 7:1420. 10.3389/fmicb.2016.01420 PMC5013055 27656179

[14] Ochoa TJ, Ruiz J, Molina M, Del Valle LJ, Vargas M, Gil AI, Ecker L, Barletta F, Hall E, Cleary TG, Lanata CF. High frequency of antimicrobial drug resistance of diarrhoegenic *Escherichia coli* in infants in Peru. Am J Trop Med Hyg. 2009;81:296–301. 19635887 PMC2824507

[15] Bezatu M, Yemane B, Alemeyehu W. Prevalence of diarrhoea and associated risk factors among children under five years of age in Eastern Ethiopia: a cross-sectional study. Open J Preview Med. 2013;37:446–53.

[16] Ali MM, Ahmed SF, Klena JD, Mohamed ZK, Moussa TA, Ghenghesh KS. Enteroaggregative *E. Coli* in diarrheic children in Egypt: molecular characterization and antimicrobial susceptibility. J Infect Developing Ctries. 2014;85:589–96. 10.3855/jidc.4077. 10.3855/jidc.4077 24820462

[17] Dudek-Wicher RK, Junka A, Bartoszewicz M. The influence of antibiotics and dietary components on gut microbiota. Przegladgastroenterologiczny. 2018;13(2):85–92. 10.5114/pg.2018.76005 PMC6040098 30002765

[18] Hill C, Guarner F, Reid G, Gibson GR, Merenstein D, Pot B, Morelli L, Canani RB, Flint H, Salminen J, Calder S, P. C., and, Sanders ME. 2014. The International Scientific Association for Probiotics and Prebiotics consensus statement on the scope and appropriate use of the term probiotic. *Nature Reviews Gastroenterology & Hepatology* 11, pp 506–514. 10.1038/nrgastro.2014.66 24912386

[19] Ayeni FA, Adeniyi BA, Ogunbanwo ST, Tabasco R, Paarup T, Peláez C, Requena T. Inhibition of uropathogens by lactic acid bacteria isolated from dairy foods and cow’s intestine in western Nigeria. Arch Microbiol. 2009;191:8, 639–48. 19529917 10.1007/s00203-009-0492-9

[20] Zhang YJ, Li S, Gan RY, Zhou T, Xu DP, Li HB. Impacts of gut bacteria on human health and diseases. Int J Mol Sci. 2015;16(4):7493–519. 25849657 10.3390/ijms16047493 PMC4425030

[21] Grover S, Rashmi HM, Srivastava AK, Batish V, K. Probiotics for human health –new innovations and emerging trends. Gut Pathogens. 2012;4:15. 10.1186/1757-4749-4-15. 23181893 10.1186/1757-4749-4-15 PMC3544614

[22] Hori T, Matsuda K, Oishi K, Probiotics. A dietary factor to modulate the gut microbiome, host Immune System, and gut–brain Interaction. Microorganisms. 2020;8:1401. 32933067 10.3390/microorganisms8091401 PMC7563712

[23] Sanders ME, Merenstein DJ, Reid G, Gibson GR, Rastall RA. Probiotics and prebiotics in intestinal health and disease: from biology to the clinic Probiotics. Nat Reviews Gastroenterol Hepatol. 2019;16(9):605–16. 10.1038/s41575-019-0173-3 31296969

[24] Moles L, Gómez M, Heilig H, Bustos G, Fuentes S, de Vos W, Fernández L, Rodríguez JM, Jiménez. E.2013.Bacterial diversity in meconium of preterm neonates and evolution of their faecal microbiota during the first month of life. PLoS One8 e66986. 10.1371/journal.pone.0066986 PMC3695978 23840569

[25] Timmerman HM, Rutten N, Boekhorst J, Saulnier DM, Kortman G, Contractor N, Kullen M, Floris E, Harmsen H, Vlieger AM, Kleerebezem M, Rijkers GT. Intestinal colonisation patterns in breastfed and formula-fed infants during the first 12 weeks of life reveal sequential microbiota signatures. Sci Rep. 2017;7(1):8327. 28827640 10.1038/s41598-017-08268-4 PMC5567133

[26] George S, Aguilera X, Gallardo P, Farfán M, Lucero Y, Torres JP, Vidal R, O’Ryan M. 2022. Bacterial Gut Microbiota and Infections During Early Childhood. Front Microbiol. 2022;12:793050. 10.3389/fmicb.2021.793050. PMID: 35069488; PMCID: PMC8767011. 10.3389/fmicb.2021.793050 PMC8767011 35069488

[27] Kamada N, Chen GY, Inohara N, Núñez G. 2013. Control of pathogens and pathobionts by the gut microbiota. Nature Immunology. 2013:14, 685–690. 10.1038/ni.2608 PMC4083503 23778796

[28] Yoon MY, Yoon SS. 2018 disruption of the gut ecosystem by antibiotics. Yonsei Med J 59:1, pp4–12. 10.3349/ymj.2018.59.1.4. PMID: 29214770; PMCID: PMC5725362. 10.3349/ymj.2018.59.1.4 PMC5725362 29214770

[29] Afolayan AO, Ayeni FA. Antagonistic effects of three lactic acid bacterial strains isolated from Nigerian indigenous fermented Ogi on *E. Coli* EKT004 in co-culture. Acta Aliment Int J Food Sci. 2017;46(1):1–8.

[30] Kwasi RE, Aremu IG, Dosunmu QO, Ayeni FA. Viability of lactic acid bacteria in different components of Ogi with anti diarrheagenic *E. Coli* activities. North Afr J food Nutr Res. 2019;03(06):206–13.

[31] Sowemimo AF, Obisesan AO, Ayeni FA. Evaluation of lactic acid bacteria viability and anti-diarrhoeagenic Escherichia coli activities of non-alcoholic fermented beverage ‘Kunu’. Croatian J Food Sci Technol. 2021;13(1):122–7. 10.17508/CJFST.2021.13.1.15.

[32] Saturio S, Nogacka AM, Alvarado-Jasso GM, Salazar N, de Los Reyes-Gavilán CG, Gueimonde M, Arboleya S. 2021. Role of Bifidobacteria on Infant Health. *Microorganisms* 23;9(12):2415. 10.3390/9122415 10.3390/microorganisms9122415 PMC8708449 34946017

[33] Medjaoui I, Rahmani B, Talhi M, Zohra F, Mahammi F, Moghtit F, Mehtar N, Bechir S, Gaouar S. Isolation and characterization of lactic acid bacteria from human milk and newborn faeces. J Pure Appl Microbiol. 2016;10(4):2613–20.

[34] Pinloche E, McEwan N, Marden JP, Bayourthe C, Auclair E, Newbold CJ. The effects of a probiotic yeast on the bacterial diversity and population structure in the rumen of cattle. PLoS ONE. 2013;87:e67824. 10.1371/journal.pone.0067824 PMC3699506 23844101

[35] Serrano-Niño JC, Solís-Pacheco JR, Gutierrez-Padilla JA, Cobián-García A, Cavazos-Garduño A, González-Reynoso O, Aguilar-Uscanga BR. 2016. Isolation and Identification of Lactic Acid Bacteria from Human Milk with Potential Probiotic Role. *Journal of Food and Nutrition Research*. Vol. 4, No. 3, 2016, pp 170–177. http://pubs.sciepub.com/jfnr/4/3/7

[36] Alebiosu KM, Adetoye A, Ayeni FA. Antimicrobial activities of lactic acid bacteria against Pseudomonas aeruginosa, Providencia vermicola, Alcaligenes faecalis and methicillin-resistant S. Aureus. West Afr J Pharm. 2017;28(2):132–42.

[37] Adetoye A, Pinloche E, Adeniyi BA, Ayeni FA. Characterization and anti-*Salmonella* activities of lactic acid bacteria isolated from cattle faeces. BMC Microbiol. 2018;18:96. 30165820 10.1186/s12866-018-1248-y PMC6118008

[38] Hassanzadazar H, Ehsani A, Mardani K, &Hesari J. Investigation of antibacterial, acid and bile tolerance properties of lactobacilli isolated from Koozeh cheese. Veterinary Res Forum: Int Q J. 2012;3(3):181–5. PMC4299980 25610566

[39] Krausova G, Hyrslova I, Hynstova I. In vitro evaluation of adhesion capacity, hydrophobicity, and auto-aggregation of newly isolated potential probiotic strains. Fermentation. 2019;5:4.

[40] Pessoa WFB, Melgaço ACC, de Almeida ME, Ramos LP, Rezende RP, Romano CC. In Vitro Activity of Lactobacilli with Probiotic potential isolated from Cocoa Fermentation against Gardnerella vaginalis. Biomed Res Int. 2017;2017(2017):3264194. 29226130 10.1155/2017/3264194 PMC5684529

[41] Chaudhuri RR, Sebaihia M, Hobman JL, Webber MA, Leyton DL, Goldberg MD, Cunningham AF, Scott-Tucker A, Ferguson PR, Thomas CM, Frankel G, Tang CM, Dudley EG, Roberts IS, Rasko DA, Pallen MJ, Parkhill J, Nataro JP, Thomson NR, Henderson IR. Complete genome sequence and comparative metabolic profiling of the prototypical enteroaggregative Escherichia coli strain 042. PLoS ONE. 2010;5(1):e8801. pmid:20098708. 20098708 10.1371/journal.pone.0008801 PMC2808357

[42] Jadhav S, Shah R, Bhave M, Palombo E. Inhibitory activity of yarrow essential oil on Listeria planktonic cells and biofilms. Food Control. 2013;29:125–30.

[43] EFSA. Technical guidance prepared by the FEEDAP Panel. Update of the criteria used in the assessment of bacterial resistance to antibiotics of human or veterinary importance. EFSA J. 2018;732:1–15. 10.2903/j.efsa.2008.732 PMC10193621 37213835

[44] EUCAST. Breakpoint tables for interpretation of MICs and zone diameters. Version 5.0. European Committee on Antimicrobial Susceptibility Testing; 2015.

[45] Halder D, Mandal M, Chatterjee SS, Pal NK, Mandal S. Indigenous probiotic *Lactobacillus* isolates presenting antibiotic like activity against human pathogenic bacteria. Biomedicines. 2017;5:2. 28621711 10.3390/biomedicines5020031 PMC5489817

[46] Lackey KA, Williams JE, Meehan CL, Zachek JA, Benda ED, Price WJ, Foster JA, Sellen DW, Kamau-Mbuthia EW, Kamundia EW, Mbugua S, Moore SE, Prentice AM, Gindola KG, Kvist LJ, Otoo GE, García-Carral C, Jiménez E, Ruiz L, Rodríguez JM, Pareja RG, Bode L, McGuire MA, McGuire MK. Corrigendum: what is normal? Microbiomes in human milk and infant faeces are related to each other but vary geographically: the INSPIRE study. Front Nutr. 2020;6:45. 10.3389/fnut.2019.00045.

[47] Stiemsma LT, Michels KB. role Microbiome Dev Origins Health Disease Pediatr. 2018;141:e20172437. 10.1542/peds.2017-2437 PMC5869344 29519955

[48] Albesharat R, Ehrmann MA, Korakli M, Yazaji S, Vogel RF. Phenotypic and genotypic analyses of lactic acid bacteria in local fermented food, breast milk and faeces of mothers and their babies. Syst Appl Microbiol. 2011;341:48–55. 10.1016/j.syapm.2010.12.001 21300508

[49] Taghizadeh M, Safaei HG, Poursina F. Identification of Lactobacillus plantarum in breast milk. Res Mol Med (RMM). 2018;5(4):50–60. 10.18502/rmm.v5i4.30.

[50] Talashi S, Sharma N. Isolation of *Lactobacillus plantarum* from humanBreast milk with probiotic and medical attributes. Acta Sci Microbiol. 2019;ISSN(2):2581–3226.

[51] Davoodabadi A, SoltanDallal MM, Lashani E, Tajabadi Ebrahimi M. 2015. Antimicrobial Activity of *Lactobacillus* spp. Isolated from Fecal Flora of Healthy Breast-Fed Infants Against Diarrheagenic Escherichia coli. Jundishapur J Microbiol. 2015;8(12):e27852. 10.5812/jjm.27852. PMID: 26865944; PMCID: PMC4745268. 10.5812/jjm.27852 PMC4745268 26865944

[52] AlKalbani NS, Turner MS, Ayyash MM. Isolation, identification, and potential probiotic characterization of isolated lactic acid bacteria and in vitro investigation of the cytotoxicity, antioxidant, and antidiabetic activities in fermented sausage. Microb Cell Fact. 2019;5:18(1):188. 10.1186/s12934-019-1239-1. PMID: 31690323; PMCID: PMC6833168. 10.1186/s12934-019-1239-1 PMC6833168 31690323

[53] Chun TG, Wang F, Chun YL, Liu F, Huo GC. 2012. *Lactobacillus xiangfangensis* sp. nov., isolated from Chinese pickle. *International Journal of Systemic and Evolutionary Microbiology* 2012; 62:860–863. 10.1099/ijs.0.031468-0 21622835

[54] Murphy K, Curley D, O’Callaghan TF, O’Shea C, Dempsey EM, O’Toole PW, Ross RP, Ryan CA, Stanton C. The Composition of Human Milk and infant faecal microbiota over the First Three months of life: a pilot study. Sci Rep. 2017;7:40597. 28094284 10.1038/srep40597 PMC5240090

[55] Jara S, Sanchez M, Vera R, Cofre J, Castro E. The inhibitory activity of *Lactobacillus* spp. isolated from breast milk on gastrointestinal pathogenic bacteria of nosocomial origin. Anaerobe. 2011;17:474–7. 21846506 10.1016/j.anaerobe.2011.07.008

[56] Zhang X, Ali Esmail G, Fahad Alzeer A, Valan Arasu M, Vijayaraghavan P, Choon Choi K, Abdullah A, N. Probiotic characteristics of Lactobacillus strains isolated from cheese and their antibacterial properties against gastrointestinal tract pathogens. Saudi J Biol Sci. 2020;27(12):3505–13. 10.1016/j.sjbs.2020.10.022. 33304162 10.1016/j.sjbs.2020.10.022 PMC7715019

[57] Abdelhamid AG, Esaam A, Hazaa MM. Cell-free preparations of probiotics exerted antibacterial and antibiofilm activities against multidrug-resistant *E. Coli*. Saudi Pharm J. 2018;265:603–7. 10.1016/j.jsps.2018.03.004 PMC6035330 29991904

[58] Kaur S, Sharma P, Kalia N, Singh J, Kaur S. Anti-biofilm properties of the feacal probiotic lactobacilli against Vibrio Spp. Front Cell Infect Microbiol. 2018;8:120. 29740541 10.3389/fcimb.2018.00120 PMC5928150

[59] Barzegari A, Kheyrolahzadeh K, HosseiniyanKhatibi SM, Sharifi S, Memar MY, ZununiVahed S. The battle of probiotics and their derivatives against biofilms. Infect drug Resist. 2020;13:659–72. 32161474 10.2147/IDR.S232982 PMC7049744

[60] Flahaut S, Hartke A, Giard JC, Benachour A, Boutibonnes P, Auffray Y. Relationship between stress response towards bile salts, acid and heat treatment in *Enterococcus faecalis*. FEMS Microbiol Letter. 1996;138:49–54. 10.1111/j.1574-6968.1996.tb08133.x 8674969

[61] van de Guchte M, Serror P, Chervaux C, Smokvina T, Ehrlich SD, Maguin E. Stress responses in lactic acid bacteria. Antonie Van Leeuwenhoek. 2002;82(1–4):187–216. PMID: 12369188. 12369188

[62] Papadimitriou K, Alegría Á, Bron PA, de Angelis M, Gobbetti M, Kleerebezem M, Lemos JA, Linares DM, Ross P, Stanton C, Turroni F, van Sinderen D, Varmanen P, Ventura M, Zúñiga M, Tsakalidou E, Kok J. Stress physiology of lactic acid Bacteria. Microbiol Mol Biol Rev. 2016;80(3):837–90. 10.1128/MMBR.00076-15. PMID: 27466284; PMCID: PMC4981675. 27466284 10.1128/MMBR.00076-15 PMC4981675

[63] Chou LS, Weimer B. Isolation and characterization of acid- and bile-tolerant isolates from strains of Lactobacillus acidophilus. J Dairy Sci. 1999;82(1):23–31. 10.3168/jds.S0022-0302(99)75204-5. PMID: 10022003. 10.3168/jds.S0022-0302(99)75204-5 10022003

[64] Food and Agriculture Organization (FAO)/WHO. Technical meeting on probiotics: food quality and standards service (AGNS). Rome, Italy: Food and Agriculture Organization of the United Nations (FAO); 2006. Sep 15–16, FAO Technical Meeting Report.

[65] Cervantes-Elizarrarás A, Cruz-Cansino NS, Ramírez-Moreno E, Vega-Sánchez V, Velázquez-Guadarrama N, Zafra-Rojas QY, Piloni-Martini J. In vitro probiotic potential of lactic acid bacteria isolated from Aguamiel and Pulque and antibacterial activity against pathogens. Appl Sci. 2019;9:601.

[66] Li M, Wang Y, Cui H, Li Y, Sun Y, Qiu HJ. (2020). Characterization of Lactic Acid Bacteria Isolated from the Gastrointestinal Tract of a Wild Boar as Potential Probiotics. Front Vet Sci. 2020;7:49. 10.3389/fvets.2020.00049. PMID: 32118070; PMCID: PMC7026679. 10.3389/fvets.2020.00049 PMC7026679 32118070

[67] Shah NP, Jelen P. Survival of lactic acid Bacteria and their lactases under acidic conditions. J Food Sci. 2006;55:506–9. 10.1111/j.1365-2621.1990.tb06797.x.

[68] Nami Y, Vaseghi Bakhshayesh R, Mohammadzadeh Jalaly H, Lotfi H, Eslami S, Hejazi MA. Probiotic properties of Enterococcus isolated from artisanal dairy products. Frontier Microbiol. 2019;10:300. 10.3389/fmicb.2019.00300. 10.3389/fmicb.2019.00300 PMC6400110 30863379

[69] Diana I, Serrazanetti D, Chiara M, Cianotti A. 2013. Dynamic stress of lactic acid bacteria associated to fermentation processes. Books: *Lactic acid Bacteria-Rand D for Food, Health and Livestock Purposes. Interchen open access peer-reviewed chapter*.

[70] Kozak K, Charbonneau D, Sanozky-Dawes R, Klaenhammer T. Characterization of bacterial isolates from the microbiota of mothers’ breast milk and their infants. Gut Microbes. 2015;6(6):341–51. 26727418 10.1080/19490976.2015.1103425 PMC4826109

[71] Puphan K, Sornplang P, Uriyapongson S, Navanukrav C. Screening of lactic acid bacteria as potential probiotics in beef cattle. Pakistan J Nutr. 2015;14:474–9.

[72] Zhang B, Wang Y, Tian Z, Li Z, Jiao Z, Huang Q. Screening of probiotic activities of lactobacilli strains isolated from traditional tibetan qual, a raw yak milk cheese. Asian-Australasian J Anim Sci. 2016;29(10):1490–9. 10.5713/ajas.15.0849 PMC5003976 26954218

[73] Garcia-Gonzalez N, Prete R, Battista N, Corsetti A. Adhesion properties of Food-Associated *Lactobacillus plantarum* strains on human intestinal epithelial cells and modulation of IL-8 release. Front Microbiol. 2018;9:2392. 30349520 10.3389/fmicb.2018.02392 PMC6186789

[74] van Zyl WF, Deane SM, Dicks LMT. Molecular insights into probiotic mechanisms of action employed against intestinal pathogenic bacteria. Gut Microbes. 2020;12(1):1831339. 10.1080/19490976.2020.1831339. PMID: 33112695; PMCID: PMC7595611. 33112695 10.1080/19490976.2020.1831339 PMC7595611

[75] Monteagudo-Mera A, Rastall RA, Gibson GR, Charalampopoulos D, Chatzifragkou A. Adhesion mechanisms mediated by probiotics, prebiotics, and their potential impact on human health. Appl Microbiol Biotechnol. 2019;10316:6463–72. 10.1007/s00253-019-09978-7 PMC6667406 31267231

[76] Tuo Y, Yu H, Ai L, Wu Z, Guo B, Chen W. Aggregation and adhesion properties of 22 Lactobacillus strains. J Dairy Sci. 2013;96(7):4252–7. 23664349 10.3168/jds.2013-6547

[77] Grajek K, Sip A, Foksowicz-Flaczyk J, Dobrowolska A, Wita A. Adhesive and hydrophobic properties of the selected LAB isolated from the gastrointestinal tract of farming animals. Acta Biochim Pol. 2016;63:2. 10.18388/abp.2015_1128 27231726

[78] Tulumoglu S, Yuksekdag ZN, Beyatli Y, Simsek O, Cinar B, Yaşar E. 2013. Probiotic properties of lactobacilli species isolated from children’s faeces. *Anaerobe*24:36–42. 10.1016/j.anaerobe.2013.09.006 24055630

[79] Lavanya B, Sowmiya S, Balaji S, Muthuvelan B. Screening and characterization of lactic acid bacteria from fermented milk. Br J Dairy Sci. 2011;2(1):5–10.

[80] Campedelli I, Mathur H, Salvetti E, Clarke S, Rea MC, Torriani S, Ross RP, Hill C, O’Toole PW. 2018. Genus-wide assessment of antibiotic resistance in *Lactobacillus* spp. Appl Environ Microbiol 85:1, e01738 - e01818. 10.1128/AEM.01738-18 PMC6293106 30366997

[81] Thumu SCR, Halami PM. Presence of erythromycin and tetracycline resistance genes in lactic acid bacteria from fermented foods of Indian origin. Antonie Van Leeuwenhoek. 2012;102:541–51. 22644346 10.1007/s10482-012-9749-4

[82] Sukmarini L, Mustopa A, Normawati M, Muzdalifah I. Identification of antibiotic-resistance genes from lactic acid bacteria in Indonisian fermented foods. HAYATI J Biosci. 2014;21:3.

[83] Drago L, Rodighiero V, Mattina R, Toscano M, DeVecchi E. In vitro selection of antibiotic resistance in the probiotic strain *Lactobacillus rhamnosus* GG ATCC 53103. J Chemother. 2011;23:4211–215. 10.1179/joc.2011.23.4.211 21803698

[84] FAO, WHO., 2002. Guidelines for the evaluation of probiotics in food. Joint FAO/WHO Workgroup on Drafting Guidelines for the Evaluation of Probiotics in Food. London, Ontario, Canada, April 30 and May 1, 2002.

[85] Asan-Ozusaglam M, Gunyakti A. *Lactobacillus fermentum* strains from human breast milk with probiotic properties and cholesterol-lowering effects. Food Sci Biotechnol. 2018;28(2):501–9. 10.1007/s10068-018-0494-y PMC6431312 30956862

[86] García A, Navarro K, Sanhueza E, Pineda S, Pastene E, Quezada M, Henríquez K, Karlyshev A, Villena J, González C. Characterization of Lactobacillus fermentum UCO-979 C, a probiotic strain with a potent anti-helicobacter pylori activity. Electron. J Biotechnol. 2017;25:75–83.

[87] Bajagai YS, Klieve AV, Dart PJ, Bryden WL. 2016. Probiotics in animal nutrition–production, impact and regulation by FAO *Animal production and health paper* No. 179; Harinder, P.S., Ed.; FAO: Rome, Italy, ISBN 978-92-5-109333-7.

[88] Mendes V, Galvão I, Vieira AT. Mechanisms by which the gut microbiota influences cytokine production and modulates host inflammatory responses. J Interferon Cytokine Res. 2019. 10.1089/jir.2019.0011. 31013453 10.1089/jir.2019.0011

[89] Kwon HK, Jo WR, Park HJ. The immune-enhancing activity of *C. Militaris* fermented with *Pediococcus pentosaceus* (GRC-ON89A) in cyclophosphamide-induced immunosuppressed model. BMC Complement Altern Med. 2018;18:1. 29475435 10.1186/s12906-018-2133-9 PMC5824477

[90] Meng Y, Li B, Jin D, Zhan M, Lu J, Huo G. Immunomodulatory activity of *Lactobacillus plantarum* KLDS1.0318 in cyclophosphamide-treated mice. Food Nutr Res. 2018;62. 10.29219/fnr.v62.1296. 10.29219/fnr.v62.1296 PMC5883861 30026678

[91] Zhou X, Dong Q, Kan X, Peng L, Xu X, Fang Y. Immunomodulatory activity of a novel polysaccharide from Lonicera japonica in immunosuppressed mice induced by cyclophosphamide. PLoS ONE. 2018;13(10):e0204152. 30296293 10.1371/journal.pone.0204152 PMC6175272

[92] Bronte V, Pittet MJ. The spleen in local and systemic regulation of immunity. Immunity. 2013;395:806–18. 10.1016/j.immuni.2013.10.010 PMC3912742 24238338

[93] Sabry A, El-Naggar AA, Alm-Eldeen MO, Germoush KF, El-Boray, Hassan AE. Ameliorative effect of propolis against cyclophosphamide-induced toxicity in mice. Pharm Biol. 2015;53(2):235–41. 25289525 10.3109/13880209.2014.914230

[94] Liu Q, Yu Z, Tian F, Zhao J, Zhang H, Zhai Q, Chen W. Surface components and metabolites of probiotics for regulation of intestinal epithelial barrier. Microb Cell Fact. 2017;19:23. 10.1186/s12934-020-1289-4 PMC7003451 32024520

[95] Kittana H, Gomes-Neto JC, Heck K, Geis AL, Segura, Muñoz RR, Cody LA, Schmaltz RJ, Bindels LB, Sinha R, Hostetter JM, Benson AK, Ramer-Tait AE. Commensal Escherichia coli strains can promote intestinal inflammation via differential Interleukin-6 production. Frontier Immunol. 2018;9:2318. 10.3389/fimmu.2018.02318. 10.3389/fimmu.2018.02318 PMC6189283 30356663

[96] Xie JH, Fan ST, Nie SP, Yu Q, Xiong T, Gong D, Xie MY. *Lactobacillus plantarum* NCU116 attenuates cyclophosphamide-induced intestinal mucosal injury, metabolism and intestinal microbiota disorders in mice. Food Function. 2016;7:1584–92. 10.1039/c5fo01516b. 10.1039/c5fo01516b 26906433

