# Supplementary material for: Lactic acid bacteria isolated from women’ breast milk and infants’ faeces have appreciable immunogenic and probiotic potentials against diarrheagenic E. coli strains
Source: BMC Microbiol. 2024 Sep 17;24:350. doi: 10.1186/s12866-024-03502-2 (PMC11406810; doi:10.1186/s12866-024-03502-2)
Supplement: Supplementary file 1 — Supplementary Material 1 [file 12866_2024_3502_MOESM1_ESM.docx]

**Supplementary Table 1.** Antimicrobial activity assay of Cell Free Supernatant of lactic acid bacteria against diarrhoeagenic *E. coli*

| Name of Isolates | Source of the Isolates | ETEC (H40B) | EPEC (H62E) | EIEC (H68D) | STEC (H77E) | EAEC (H40C) |
| --- | --- | --- | --- | --- | --- | --- |
| *L. plantarum* A002 | Breast Milk | 20.37 ±0.81 | 0.00 ± 0.00 | 8.30 ± 0.17 | 13.23±0.40 | 9.00±0.20 |
| *E. faecium* A003 | Breast Milk | 16.37±1.01 | 10.33±0.58 | 11.00 ± 0.50 | 13.07±0.12 | 14.00±0.60 |
| *E. faecium* A008 | Breast Milk | 14.00±1.00 | 8.10±0.10 | 0.00 ± 0.00 | 16.13±0.23 | 15.00±0.20 |
| *L. pseudomenseteroides*A010 | Breast milk | 16.10±1.30 | 7.00 ± 0.00 | 8.13±0.15 | 18.13±0.15 | 14.10±0.10 |
| *L. plantarum* A011 | Faeces | 15.23±0.20 | 12.30±0.20 | 10.27±0.12 | 21.00±0.10 | 18.17±0.40 |
| *L. rhamnosus* A012 | Faeces | 24.20±1.00 | 10.13±0.81 | 13.93 ± 0.15 | 17.20±0.20 | 16.10±0.10 |
| *E. durans*A013 | Faeces | 24.17±0.40 | 12.47±0.12 | 8.30 ±0.06 | 17.17±0.21 | 16.2±0.36 |
| *L. plantarum* A014 | Faeces | 12.20±0..20 | 14.07±0.23 | 14.20± 0.17 | 14.20±0.35 | 18.23±0.32 |
| *L. pentosus*A016 | Breast Milk | 14.43±0.67 | 11.49±0.57 | 0.00 ± 0.00 | 18.13±0.12 | 15.03±0.21 |
| *L. paracasei*A017 | Breast Milk | 18.03±0.12 | 0.00 ± 0.00 | 0.00 ± 0.00 | 15.13±0.23 | 16.17±0.15 |
| *E. faecium* A018 | Breast Milk | 9.30±0.20 | 12.10±0.70 | 10.30±0.17 | 19.13±0.15 | 16.57±0.74 |
| *L. pseudomenseteroides*A019 | Breast Milk | 14.07±0.35 | 0.00 ± 0.00 | 0.00 ± 0.00 | 21.20±0.10 | 19.10±0.30 |
| *E. faecium* A022 | Breast Milk | 13.60±0.40 | 0.00 ± 0.00 | 10.07±0.12 | 14.10±0.10 | 14.10±0.10 |
| *L. plantarum* A023 | Feaces | 21.20±0.20 | 12.13±0.06 | 14.17±0.12 | 16.30±0.10 | 11.20±0.20 |
| *E. durans*A024 | Feaces | 16.20±0..20 | 12.03±0.45 | 12.07±0.12 | 19.17±0.21 | 20.00±0.20 |
| *L. pseudomenseteroides*A026 | Faeces | 12.20±0.20 | 13.20±0.60 | 14.13±0.15 | 14.17±0.06 | 16.03±0.21 |
| *E. .thailandicus*A027 | Faeces | 10.10±0.46 | 16.00±0.50 | 10.93±0.12 | 12.17±0.12 | 21.00±0.20 |
| *L. pentosus*A028 | Breast Milk | 16.10±0.00 | 16.20±0.20 | 10.07±0.12 | 16.13±0.15 | 13.10±0.10 |
| *L. pseudomenseteroides*A030 | Feaces | 15.30±0.20 | 0.00 ± 0.00 | 0.00 ± 0.00 | 12.20 ± 0.20 | 16.00±0.00 |
| *E. thailandicus*A031 | Faeces | 10.23±0.20 | 0.00 ± 0.00 | 0.00 ± 0.00 | 14.23±0.15 | 12.23±0.15 |
| *L. plantarum* A033 | Breast Milk | 10.30±0.20 | 0.00 ± 0.00 | 0.00 ± 0.00 | 21.13±0.12 | 16.17±0.12 |
| *L. plantarum* A034 | Breast Milk | 16.03±0.25 | 10.17±0.15 | 12.1±0.17 | 18.23±0.23 | 18.17±0.21 |
| *L. plantarum* A035 | Breast Milk | 12.10±0.00 | 16.23±0.21 | 14.2±0.17 | 17.07±0.12 | 18.00±0.10 |
| *L. plantarum* A036 | Breast Milk | 15.10±0.10 | 12.07±0.12 | 0.00 ± 0.00 | 16.10±0.10 | 12.10± 0.30 |
| *L. plantarum* A037 | Breast Milk | 13.30±0.20 | 0.00 ± 0.00 | 0.00 ± 0.00 | 16.13±0.15 | 15.13± 0.31 |
| *E. durans* A038 | Breast Milk | 11.17±0.21 | 13.70±0.32 | 0.00 ± 0.00 | 14.20±0.20 | 14.03±0.21 |
| *E. faecium* A039 | Breast Milk | 24.10±0.10 | 18.07±0.06 | 10.17±0.12 | 21.03±0.25 | 16.50 ± 0.10 |
| *E. faecium* A040 | Breast Milk | 18.13±0.23 | 14.03±0.21 | 12.40 ± 0.10 | 14.20±0.20 | 18.38±0.12 |

**Supplementary Table 1 cont.**

| Name of Isolates | Source of the Isolates | ETEC (H40B) | EPEC (H62E) | EIEC (H68D) | STEC (H77E) | EAEC (H40C) |
| --- | --- | --- | --- | --- | --- | --- |
| *L. plantarum* A041 | Breast Milk | 19.17±0.12 | 0.00 ± 0.00 | 0.00 ± 0.00 | 18.13±0.15 | 16.30±0.10 |
| *E. durans* A043 | Breast Milk | 12.23±0.25 | 0.00 ± 0.00 | 0.00 ± 0.00 | 14.20±0.35 | 8.13±0.23 |
| *L. pseudomenseteroides* A044 | Breast Milk | 16.23±0.25 | 12.27±0.23 | 13.17± 0.06 | 17.07±0.06 | 19.20±0.20 |
| *L. plantarum* A046 | Breast Milk | 20.17±0.21 | 16.37±0.12 | 10.43±0.12 | 15.10±0.1 | 19.07±0.4 |
| *L. plantarum* A047 | Breast Milk | 19.23±0.25 | 17.17±0.21 | 0.00 ± 0.00 | 14.2± 0.27 | 10.6±0.20 |
| *L. plantarum* A048 | Breast Milk | 15.00±0.15 | 14.30 ± 0.30 | 0.00 ± 0.00 | 10.17±0.29 | 11.97±0.15 |
| *E. faecium* A049 | Breast Milk | 12.10±0.10 | 0.00 ± 0.00 | 0.00 ± 0.00 | 16.27±0.25 | 16.00±0.20 |
| *E. durans*A050 | Breast Milk | 21.10±0.10 | 14.20±0.20 | 14.23±0.15 | 16.20±0.27 | 18.17±0.35 |
| *L. plantarium*A051 | Breast Milk | 18.10±0.17 | 14.13±0.12 | 8.33±0.06 | 21.17±0.32 | 18.10±0.50 |
| *E. lactis* A052 | Breast Milk | 10.13±0.23 | 12.07±0.55 | 0.00 ± 0.00 | 19.20±0.17 | 21.03±0.21 |
| *E. faecalis* A058 | Faeces | 14.07±0.31 | 0.00 ± 0.00 | 0.00 ± 0.00 | 0.00 ± 0.0 | 21.00±0.20 |
| *L. plantarium*A059 | Faeces | 23.10±0.10 | 10.07±0.25 | 10.20±0.34 | 22.20±0.27 | 23.40±0.61 |
| *E. lactis* A060 | Faeces | 13.17±0.15 | 0.00 ± 0.00 | 0.00 ± 0.00 | 11.13±0.15 | 14.30±0.15 |
| *L. pseudomesenteroides* A064 | Faeces | 21.13±0.35 | 12.03± 0.55 | 10.27±0.31 | 16.23±0.21 | 22.10± 1.00 |
| *E. faecium* A066 | Faeces | 20.13±0.15 | 11.13±0.12 | 12.37±0.15 | 20.10±0.10 | 12.30± 0.10 |
| *L. plantarum* A071 | Breast Milk | 13.17±0.25 | 12.23±0.25 | 0.00 ± 0.00 | 11.27±0.12 | 13.24 ±0.15 |
| *L. rhamnosus* A072 | Breast Milk | 20.30±0.30 | 16.33±0.21 | 10.23±0.12 | 17.17±0.29 | 16.10±0.30 |
| *P. pentosaceus*A074 | Breast Milk | 21.13±0.15 | 15.00 ± 0.20 | 10.3±0.17 | 22.2±0.17 | 23.07±0.06 |
| *L. plantarum* A075 | Breast Milk | 16.03±0.45 | 17.00±0.50 | 11.12±0.08 | 16.17±0.15 | 16.07±0.12 |
| *E. faecalis* A077 | Faeces | 25.10±0.10 | 14.13±0.12 | 8.20±0.20 | 21.13±0.15 | 18.20±0.20 |
| *L. plantarum* A079 | Faeces | 13.20±0.20 | 10.00±0.17 | 0.00 ± 0.00 | 19.13±0.15 | 16.13±0.15 |
| *E. faecium* A080 | Breast Milk | 18.13±0.15 | 15.20±0.40 | 8.00 ±0.10 | 20.20 ± 0.20 | 20.10 ± 1.02 |
| *E. lactis* A081 | Breast Milk | 21.07±0.25 | 14.23± 0.12 | 0.00 ± 0.00 | 23.17±0.20 | 14.17± 0.21 |
| *L. pseudomesenteroides* A082 | Breast Milk | 16.10±0.10 | 13.97±0.15 | 8.00 ± 0.00 | 14.23±0.25 | 12.00 ± 1.00 |
| *E. faecium* A083 | Breast Milk | 21.20±0.20 | 15.03± 0.06 | 10.23±0.32 | 20.20±0.20 | 18.07 ±0.12 |
| *L. plantarum* A084 | Breast Milk | 14.10±0.10 | 10.00± 0.17 | 12.10±0.10 | 19.13±0.15 | 11.00 ±0.40 |
| *E. faecium* A087 | Faeces | 18.03±0.25 | 16.00 ±0.00 | 10.20±0.27 | 20.03±0.06 | 19.10± 0.10 |
| *L. pseudomesenteroides* A089 | Faeces | 14.00±0.20 | 12.07± 0.12 | 14.00±0.40 | 14.13±0.23 | 16.10± 0.10 |

**Supplementary Table 1 cont.**

| Name of Isolates | Source of the Isolates | ETEC (H40B) | EPEC (H62E) | EIEC (H68D) | STEC (H77E) | EAEC (H40C) |
| --- | --- | --- | --- | --- | --- | --- |
| *E. faecium* A090 | Faeces | 19.03±0.06 | 10.13±0.12 | 0.00 ± 0.00 | 23.10±0.10 | 21.47±1.52 |
| *L. pseudomesenteroides* A093 | Breast Milk | 26.07 ± 0.12 | 16.13±0.23 | 7.00 ± 0.00 | 10.07±0.12 | 11.00± 0.50 |
| *L. plantarium*A094 | Faeces | 10.02 ± 0.37 | 14.00±0.20 | 0.00 ± 0.00 | 14.10±0.10 | 11.20 ± 0.20 |
| *E. durans* A095 | Faeces | 21.0±0.17 | 17.00±0.20 | 0.00 ± 0.00 | 16.20±0.20 | 23.07± 0.06 |
| *E. lactis* A096 | Faeces | 14.00±0.57 | 0.00 ± 0.00 | 0.00 ± 0.00 | 14.07±0.12 | 12.10± 0.10 |
| *E. durans* A097 | Faeces | 18.17±0.86 | 12.07±0.12 | 0.00 ± 0.00 | 17.13±0.23 | 19.00 ± 0.20 |
| *E. durans* A098 | Faeces | 12.05±0.19 | 17.00± 1.00 | 10.06±0.05 | 17.00±0.06 | 13.03±0.55 |
| *E. faecalis* A4D | Faeces | 14.17±0.15 | 12.17±0.29 | 12.13±0.15 | 8.07±0.12 | 14.03±0.06 |
| *W. cibaria* B3b | Breast Milk | 10.00 ±0.15 | 10.03±0.06 | 0.00 ± 0.00 | 0.00 ± 0.00 | 10.07±0.12 |
| *L. pentosus*A1d | Faeces | 8.13±0.23 | 8.00 ± 0.00 | 0.00 ± 0.00 | 0.00 ± 0.00 | 0.00 ± 0.00 |
| *W. cibaria*B3a | Breast Milk | 10.00±0.23 | 12.07 ± 0.12 | 14.03 ±0.31 | 14.10±0.10 | 12.07± 0.12 |
| *L. fermentum* A3b | Faeces | 0.00 ± 0.0 | 0.00 ± 0.00 | 8.00 ± 0.00 | 8.07±0.12 | 0.00 ± 0.00 |
| *L. plantarum* B3c | Breast Milk | 16.10±0.03 | 8.03±0.06 | 14.13 ±0.42 | 14.17±0.29 | 10.03±0.06 |
| *L .plantarum* A4b | Faeces | 13.17±0.15 | 12.07±0.12 | 8.07±0.12 | 10.17±0.29 | 12.00 ± 0.80 |
| *L. plantarum* B1b2 | Breast Milk | 14.07±0.31 | 0.00 ± 0.00 | 17.90±0.36 | 0.00 ± 0.00 | 17.03± 0.95 |
| *L. plantarum* A1c | Faeces | 13.13±0.15 | 12.00±0.00 | 15.20±0.27 | 13.07±0.31 | 9.03±0.06 |
| *W. cibaria*B4a | Breast Milk | 15.10±0.10 | 0.00 ± 0.00 | 12.23±0.20 | 0.00 ± 0.00 | 0.00 ± 0.00 |
| *L. pentosus*A4f | Faeces | 12.10± 0.17 | 0.00 ± 0.00 | 10.17±0.21 | 16.33±0.31 | 14.07± 0.9 |
| *W. cibaria*B1e | Breast Milk | 12.23±0.25 | 0.00 ± 0.00 | 0.00 ± 0.00 | 0.00 ± 0.00 | 8.07±0.06 |
| *L. fermentum* A3a | Faeces | 8.13±0.23 | 0.00 ± 0.00 | 14.27±0.31 | 13.10±0.10 | 10.00 ± 0.00 |
| *W. cibaria*B4c | Breast Milk | 8.13±0.23 | 0.00 ± 0.00 | 0.00 ± 0.00 | 0.00 ± 0.00 | 0.00 ± 0.00 |
| *L. pentosus* A4c | Faeces | 15.13±0.15 | 14.03±0.06 | 13.17±0.15 | 15.20 ± 0.20 | 14.13±0.91 |
| *W. confuse* B1d | Breast Milk | 8.13±0.06 | 0.00 ± 0.00 | 0.00 ± 0.00 | 0.00 ± 0.00 | 0.00 ± 0.00 |
| *L. pentosus* A1a | Faeces | 8.07±0.12 | 8.00 ±0..00 | 10.01±0.1 | 14.27 ±0.31 | 15.00 ±0.00 |
| *L. plantarum* B1f | Breast Milk | 0.00 ± 0.00 | 0.00 ± 0.00 | 8.03±0.06 | 8.03±0.06 | 7.00 ± 0.00 |
| *L. fermentum* A3d | Faeces | 0.00 ± 0.00 | 0.00 ± 0.00 | 8.10±0.17 | 0.00 ± 0.00 | 7.07 ± 0.12 |
| *L. plantarum* B1c | Breast Milk | 10.20±0.20 | 10.13±0.23 | 16.13±0.23 | 9.13±0.23 | 16.10±0.10 |
| *L. fermentum* A3c | Faeces | 0.00 ± 0.00 | 14.07±0.60 | 12.20±0.35 | 11.07±0.00 | 8.13±0.23 |
| *L. pentosus*B1b | Breast Milk | 12.03±0.06 | 17.27±0.64 | 26.10±0.56 | 26.1±0.10 | 14.00 ± 0.00 |
| *L. pentosus*A1e | Faeces | 10.03±0.25 | 0.00 ± 0.00 | 20.21±0.26 | 15.20±0.20 | 14.00 ± 0.20 |
| *L. plantarum* B1a | Breast Milk | 14.10±0.10 | 0.00 ± 0.00 | 12.07±0.12 | 13.07±0.12 | 14.00 ± 2.00 |
| *L. xianqfangensis* B1a2 | Breast Milk | 0.00 ± 0.00 | 12.13±0.23 | 12.10±0.10 | 0.00 ± 0.00 | 11.07 ± 1.00 |

**Supplementary Table 1 cont.**

| **Name of Isolates** | **Source of the Isolates** | **ETEC (H40B)** | **EPEC (H62E)** | **EIEC (H68D)** | **STEC (H77E)** | **EAEC (H40C)** |
| --- | --- | --- | --- | --- | --- | --- |
| *E. durans* A004 | Breast Milk | 0.00 ± 0.00 | 8.00 ± 0.00 | 0.00 ± 0.00 | 16.17±0.29 | 14.00 ± 0.20 |
| *E. faecalis* A020 | Breast Milk | 18.20±0.20 | 12.00±0.00 | 10.03±0.06 | 15.13±0.15 | 16.00 ± 0.50 |
| *E. faecalis* A029 | Breast Milk | 18.10±0.17 | 15.17±0.29 | 8.10 ± 0.17 | 18.17±0.15 | 16.00 ± 1.00 |
| *E. faecalis* A063 | Faeces | 0.00 ± 0.00 | 0.00 ± 0.00 | 0.00 ± 0.00 | 16.10±0.10 | 0.00 ± 0.00 |
| *E. faecalis* A078 | Faeces | 18.17±0.29 | 0.00 ±0.00 | 0.00 ± 0.00 | 0.00 ± 0.00 | 21.00 ± 0.00 |

Note: The diameter of the zone of inhibition of LAB is in mm

**Supplementary Table 2.** Tolerance of lactic acid bacteria to acid and bile

| LAB Isolates | pH3 (Log of cfu//ml) | | | | pH2(Log of cfu//ml) | | | | 0.3 % Bile(Log of cfu//ml) | | | |
| --- | --- | --- | --- | --- | --- | --- | --- | --- | --- | --- | --- | --- |
|  | Control | Initial | Final | Survival% | Control | Initial | Final | Survival% | Control | Initial | Final | Survival% |
| *L. plantarum* A002 | 8.35 ± 0.03 | 8.32 ± 0.04 | 7.92 ± 0.12 | 95.25 | 8.41 ± 0.02 | 7.88 ± 0.01 | 4.89 ± 0.07 | 62.07 | 8.40 ± 0.06 | 8.37 ± 0.03 | 8.43 ± 0.02 | 100.50 |
| *E. faecium* A003 | 8.34 ± 0.08 | 8.17 ± 0.05 | 7.33 ± 0.06 | 89.73 | 8.30 ± 0.04 | 8.01 ± 0.01 | 5.04 ± 0.04 | 62.94 | 8.17 ± 0.02 | 8.10 ± 0.05 | 7.39 ± 0.02 | 91.27 |
| *E* .faecium A008 | 8.47 ± 0.02 | 8.04 ± 0.10 | 7.25 ± 0.03 | 90.17 | 8.34 ± 0.02 | 8.05 ± 0.01 | 5.22 ± 0.06 | 64.90 | 8.02 ± 0.02 | 8.03 ± 0.03 | 7.09 ± 0.01 | 88.20 |
| *L. pseudomenseteroides* A010 | 9.23 ± 0.08 | 8.91 ± 0.02 | 8.68 ± 0.03 | 97.39 | 9.20 ± 0.05 | 8.33 ± 0.01 | 6.01 ± 0.00 | 72.14 | 8.32 ± 0.05 | 8.33 ± 0.03 | 8.22 ± 0.01 | 98.68 |
| *L. plantarum* A011 | 8.52 ± 0.03 | 8.42 ± 0.04 | 8.30 ± 0.02 | 98.59 | 8.36 ± 0.04 | 8.05 ± 0.01 | 5.22 ± 0.03 | 64.83 | 8.30 ± 0.02 | 8.08 ± 0.04 | 8.20 ± 0.01 | 101.50 |
| *L. rhamnosus* A012 | 9.26 ± 0.03 | 8.86 ± 0.11 | 7.75 ± 0.02 | 87.47 | 9.24 ± 0.04 | 8.23 ± 0.02 | 4.87 ± 0.03 | 59.24 | 8.48 ± 0.01 | 8.36 ± 0.04 | 8.46 ± 0.02 | 101.25 |
| *E. durans* 013 | 8.54 ± 0.08 | 7.71 ± 0.12 | 7.13 ± 0.02 | 92.41 | 8.34 ± 0.04 | 8.31 ± 0.01 | 0.00 ± 0.00 | 0.00 | 8.18 ± 0.02 | 8.06 ± 0.06 | 7.77 ± 0.11 | 96.50 |
| *L. plantarum* A014 | 9.12 ± 0.02 | 8.36 ± 0.03 | 8.37 ± 0.03 | 100.10 | 9.06 ± 0.11 | 8.26 ± 0.01 | 5.55 ± 0.06 | 67.22 | 8.44 ± 0.01 | 8.39 ± 0.04 | 6.21 ± 0.02 | 74.01 |
| *L. pentosus* A016 | 8.76 ± 0.06 | 8.43 ± 0.09 | 8.46 ± 0.01 | 100.39 | 8.14 ± 0.05 | 8.01 ± 0.01 | 6.02 ± 0.02 | 75.17 | 8.22 ± 0.00 | 8.11 ± 0.10 | 8.34 ± 0.03 | 102.84 |
| *L. paracasei* A017 | 8.47 ± 0.07 | 8.41 ± 0.04 | 8.35 ± 0.03 | 99.27 | 8.22 ± 0.03 | 7.98 ± 0.01 | 5.06 ± 0.06 | 63.43 | 8.44 ± 0.02 | 8.40 ± 0.03 | 8.32 ± 0.01 | 99.13 |
| *E. faecium* A018 | 9.18 ± 0.01 | 8.40 ± 0.05 | 7.23 ± 0.02 | 86.02 | 8.13 ± 0.05 | 8.02 ± 0.01 | 5.53 ± 0.06 | 68.93 | 8.05 ± 0.03 | 8.04 ± 0.04 | 7.87 ± 0.06 | 97.85 |
| *L. pseudomenseteroides* A019 | 8.45 ± 0.09 | 8.17 ± 0.09 | 7.98 ± 0.02 | 97.66 | 8.16 ± 0.05 | 8.02 ± 0.01 | 5.06 ± 0.08 | 63.09 | 7.97 ± 0.04 | 7.54 ± 0.03 | 7.09 ± 0.09 | 93.92 |
| *E. faecium* A022 | 9.21 ± 0.01 | 8.65 ± 0.02 | 8.04 ± 0.03 | 93.03 | 9.11 ± 0.13 | 8.18 ± 0.01 | 0.00 ± 0.00 | 0.00 | 8.32 ± 0.01 | 8.34 ± 0.04 | 8.04 ± 0.04 | 96.42 |
| *L. plantarum* A023 | 8.40 ± 0.18 | 8.18 ± 0.07 | 8.66 ± 0.02 | 105.86 | 8.61 ± 0.02 | 8.11 ± 0.01 | 4.79 ± 0.11 | 59.14 | 8.15 ± 0.03 | 8.09 ± 0.05 | 0.00 ± 0.00 | 0.00 |
| *E. durans* A024 | 9.30 ± 0.01 | 8.61 ± 0.03 | 8.38 ± 0.03 | 97.39 | 8.33 ± 0.51 | 8.05 ± 0.01 | 0.00 ± 0.00 | 0.00 | 8.15 ± 0.02 | 8.17 ± 0.05 | 7.83 ± 0.07 | 95.92 |
| *L. pseudomenseteroides* A026 | 8.53 ± 0.07 | 8.38 ± 0.05 | 8.19 ± 0.02 | 97.74 | 9.04 ± 0.04 | 8.84 ± 0.01 | 0.00 ± 0.00 | 0.00 | 8.37 ± 0.01 | 8.35 ± 0.05 | 8.05 ± 0.04 | 96.45 |
| *E. thailandicus* A027 | 8.46 ± 0.16 | 8.03 ± 0.09 | 7.84 ± 0.05 | 97.64 | 8.13 ± 0.04 | 7.08 ± 0.01 | 5.54 ± 0.04 | 78.33 | 8.03 ± 0.02 | 7.98 ± 0.02 | 0.00 ± 0.00 | 0.00 |
| *L. pentosus* A028 | 9.24 ± 0.01 | 8.32 ± 0.05 | 8.26 ± 0.02 | 99.33 | 9.08 ± 0.03 | 8.21 ± 0.01 | 6.06 ± 0.02 | 73.75 | 8.48 ± 0.01 | 8.46 ± 0.02 | 8.40 ± 0.02 | 99.25 |
| *L. pseudomenseteroides* A030 | 8.88 ± 0.04 | 8.22 ± 0.06 | 7.15 ± 0.03 | 86.89 | 8.43 ± 0.01 | 8.36 ± 0.01 | 4.86 ± 0.09 | 58.11 | 8.15 ± 0.02 | 8.08 ± 0.03 | 8.02 ± 0.01 | 99.23 |
| *E. thailandicus* A031 | 9.28 ± 0.01 | 8.17 ± 0.05 | 7.83 ± 0.03 | 95.78 | 8.22 ± 0.03 | 8.06 ± 0.01 | 0.00 ± 0.00 | 0.00 | 8.18 ± 0.02 | 8.05 ± 0.04 | 8.82 ± 0.10 | 109.55 |

Mean±SD

Supplementary Table 2 cont’d

| LAB Isolates | pH3 (Log10 of cfu//ml) | | | | pH2 (Log10 of cfu//ml) | | | | 0.3 % Bile (Log10 of cfu//ml) | | | |
| --- | --- | --- | --- | --- | --- | --- | --- | --- | --- | --- | --- | --- |
|  | Control | Initial | Final | Survival% | Control | Initial | Final | Survival% | Control | Initial | Final | Survival% |
| *L. plantarum* A033 | 8.76 ± 0.06 | 8.18 ± 0.05 | 7.51 ± 0.03 | 91.91 | 8.86 ± 0.01 | 8.18 ± 0.01 | 0.00 ± 0.00 | 0.00 | 8.18 ± 0.03 | 8.03 ± 0.02 | 6.69 ± 0.08 | 83.32 |
| *Lac. plantarum* A034 | 8.62 ± 0.12 | 8.26 ± 0.03 | 7.00 ± 0.05 | 84.79 | 8.39 ± 0.02 | 8.27 ± 0.01 | 4.49 ± 0.01 | 54.34 | 8.43 ± 0.02 | 8.26 ± 0.02 | 8.22 ± 0.04 | 99.58 |
| *L. plantarum* A035 | 8.60 ± 0.16 | 8.38 ± 0.06 | 8.19 ± 0.02 | 97.73 | 8.50 ± 0.04 | 8.09 ± 0.01 | 0.00 ± 0.00 | 0.00 | 8.48 ± 0.01 | 8.44 ± 0.02 | 8.23 ± 0.03 | 97.49 |
| *L. plantarum* A036 | 8.57 ± 0.04 | 8.85 ± 0.11 | 8.86 ± 0.03 | 100.13 | 8.35 ± 0.03 | 8.09 ± 0.01 | 5.76 ± 0.06 | 71.25 | 8.35 ± 0.02 | 8.24 ± 0.03 | 8.32 ± 0.02 | 100.97 |
| *L. plantarum* A037 | 9.21 ± 0.02 | 8.30 ± 0.03 | 8.29 ± 0.01 | 99.84 | 8.40 ± 0.02 | 7.99 ± 0.00 | 5.34 ± 0.04 | 66.84 | 8.51 ± 0.01 | 8.32 ± 0.02 | 8.36 ± 0.01 | 100.50 |
| *E. durans* A038 | 8.71 ± 0.04 | 8.59 ± 0.08 | 8.38 ± 0.04 | 97.48 | 8.47 ± 0.02 | 8.14 ± 0.02 | 0.00 ± 0.00 | 0.00 | 8.20 ± 0.03 | 8.09 ± 0.05 | 8.38 ± 0.03 | 103.64 |
| *E. faecium* A039 | 8.50 ± 0.15 | 8.12 ± 0.04 | 7.80 ± 0.02 | 96.06 | 8.17 ± 0.03 | 7.84 ± 0.02 | 4.79 ± 0.10 | 61.14 | 8.33 ± 0.01 | 8.36 ± 0.02 | 8.47 ± 0.01 | 101.38 |
| *E. faecium* A040 | 9.12 ± 0.01 | 8.01 ± 0.07 | 0.00 ± 0.00 | 0.00 | 8.76 ± 0.61 | 7.50 ± 0.05 | 0.00 ± 0.00 | 0.00 | 8.04 ± 0.02 | 8.05 ± 0.06 | 7.82 ± 0.07 | 97.07 |
| *L. plantarum* A041 | 8.79 ± 0.02 | 8.57 ± 0.03 | 8.33 ± 0.04 | 97.15 | 8.78 ± 0.01 | 7.91 ± 0.03 | 0.00 ± 0.00 | 0.00 | 8.46 ± 0.01 | 8.04 ± 0.04 | 8.33 ± 0.02 | 103.68 |
| *E. durans* A043 | 8.34 ± 0.08 | 8.02 ± 0.09 | 6.99 ± 0.03 | 87.14 | 9.03 ± 0.05 | 8.33 ± 0.02 | 0.00 ± 0.00 | 0.00 | 8.03 ± 0.03 | 8.03 ± 0.04 | 7.74 ± 0.07 | 96.33 |
| *L. pseudomenseteroides* A044 | 8.22 ± 0.09 | 8.20 ± 0.08 | 7.24 ± 0.02 | 88.25 | 8.25 ± 0.03 | 8.14 ± 0.03 | 4.08 ± 0.35 | 50.07 | 8.33 ± 0.02 | 8.34 ± 0.03 | 8.37 ± 0.01 | 100.37 |
| *L. plantarum* A046 | 8.44 ± 0.17 | 8.26 ± 0.05 | 8.11 ± 0.02 | 98.13 | 8.22 ± 0.03 | 8.15 ± 0.03 | 5.10 ± 0.07 | 62.54 | 8.34 ± 0.01 | 8.30 ± 0.01 | 8.04 ± 0.03 | 96.86 |
| *L. plantarum* A047 | 8.54 ± 0.04 | 8.47 ± 0.04 | 8.38 ± 0.01 | 98.98 | 8.13 ± 0.03 | 7.05 ± 0.02 | 5.32 ± 0.02 | 75.51 | 8.43 ± 0.01 | 8.38 ± 0.03 | 8.39 ± 0.02 | 100.07 |
| *L. plantarum* A048 | 8.66 ± 0.08 | 8.28 ± 0.04 | 8.20 ± 0.01 | 99.08 | 8.69 ± 0.01 | 8.49 ± 0.02 | 5.55 ± 0.04 | 65.41 | 8.25 ± 0.04 | 8.03 ± 0.03 | 8.03 ± 0.02 | 99.97 |
| *E. faecium* A049 | 8.72 ± 0.04 | 8.23 ± 0.03 | 8.05 ± 0.05 | 97.74 | 8.79 ± 0.01 | 8.11 ± 0.03 | 0.00 ± 0.00 | 0.00 | 8.25 ± 0.01 | 8.13 ± 0.02 | 0.00 ± 0.00 | 0.00 |
| *E. durans* A050 | 8.38 ± 0.11 | 8.67 ± 0.01 | 7.23 ± 0.07 | 83.36 | 8.24 ± 0.03 | 8.06 ± 0.04 | 0.00 ± 0.00 | 0.00 | 8.07 ± 0.01 | 8.04 ± 0.07 | 7.12 ± 0.01 | 88.61 |
| *L. plantarum* A051 | 8.46 ± 0.08 | 9.00 ± 0.02 | 7.14 ± 0.03 | 79.41 | 8.22 ± 0.02 | 8.04 ± 0.03 | 0.00 ± 0.00 | 0.00 | 7.98 ± 0.01 | 7.54 ± 0.05 | 0.00 ± 0.00 | 0.00 |
| *E. lactis* A052 | 9.05 ± 0.04 | 8.43 ± 0.03 | 8.25 ± 0.01 | 97.84 | 8.61 ± 0.01 | 8.04 ± 0.04 | 5.32 ± 0.02 | 66.18 | 8.08 ± 0.02 | 8.16± 0.02 | 7.86 ± 0.11 | 96.26 |
| *E. faecalis* A058 | 8.27 ± 0.07 | 8.03 ± 0.10 | 7.37 ± 0.04 | 91.78 | 9.18 ± 0.02 | 8.46 ± 0.02 | 0.00 ± 0.00 | 0.00 | 8.27 ± 0.01 | 8.14 ± 0.02 | 8.04 ± 0.04 | 98.74 |
| *L. plantarum* A059 | 9.27 ± 0.02 | 8.29 ± 0.06 | 8.19 ± 0.01 | 98.84 | 8.53 ± 0.01 | 8.26 ± 0.04 | 0.00 ± 0.00 | 0.00 | 8.29 ± 0.01 | 8.29 ± 0.02 | 8.02 ± 0.02 | 96.74 |

Mean±SD

Supplementary Table 2 cont’d

| LAB Isolates | | | pH3 (Log of cfu//ml) | | | | pH2 (Log of cfu//ml) | | | | 0.3 % Bile (Log of cfu//ml) | | | |
| --- | --- | --- | --- | --- | --- | --- | --- | --- | --- | --- | --- | --- | --- | --- |
|  | Control | | Initial | Final | Survival% | Control | Initial | Final | Survival% | Control | Initial | Final | Survival% |  |
| *E. lactis* A060 | 9.04 ± 0.01 | | 8.23 ± 0.04 | 7.93 ± 0.00 | 96.37 | 9.02 ± 0.01 | 8.32 ± 0.02 | 0.00 ± 0.00 | 0.00 | 8.08 ± 0.02 | 8.06 ± 0.02 | 7.57 ± 0.08 | 93.91 |  |
| *L. pseudomenseteroides* A064 | 9.46 ± 0.00 | | 8.70 ± 0.04 | 8.51 ± 0.01 | 97.81 | 8.47 ± 0.02 | 8.39 ± 0.04 | 5.51 ± 0.03 | 65.66 | 8.38 ± 0.00 | 8.35 ± 0.05 | 8.37 ± 0.02 | 100.27 |  |
| *E. faecium* A066 | 8.39 ± 0.05 | | 8.27 ± 0.06 | 7.18 ± 0.03 | 86.80 | 8.46 ± 0.02 | 8.33 ± 0.01 | 5.30 ± 0.12 | 63.62 | 8.26 ± 0.01 | 8.27 ± 0.02 | 7.53 ± 0.07 | 90.98 |  |
| *L. plantarum* A071 | 8.53 ± 0.03 | | 8.19 ± 0.03 | 8.09 ± 0.01 | 98.75 | 8.34 ± 0.03 | 7.98 ± 0.01 | 0.00 ± 0.00 | 0.00 | 8.21 ± 0.02 | 8.14 ± 0.03 | 7.86 ± 0.10 | 96.50 |  |
| *L. rhamnosus* A072 | 8.62 ± 0.15 | | 8.47 ± 0.04 | 8.41 ± 0.06 | 99.20 | 8.19 ± 0.03 | 8.16 ± 0.02 | 6.29 ± 0.01 | 77.12 | 8.78 ± 0.56 | 8.41 ± 0.02 | 8.37 ± 0.03 | 99.62 |  |
| *P. pentosaceus* A074 | 8.19 ± 0.04 | | 8.45 ± 0.02 | 8.38 ± 0.03 | 99.27 | 8.10 ± 0.04 | 7.48 ± 0.07 | 5.11 ± 0.07 | 68.34 | 8.47 ± 0.01 | 8.48 ± 0.01 | 8.45 ± 0.03 | 99.69 |  |
| *L. plantarum* A075 | 8.20 ± 0.10 | | 8.10 ± 0.04 | 7.37 ± 0.03 | 90.95 | 8.31 ± 0.02 | 8.26 ± 0.02 | 4.48 ± 0.00 | 54.20 | 8.13 ± 0.02 | 8.13 ± 0.03 | 8.08 ± 0.03 | 99.47 |  |
| *E. faecalis* A077 | 9.04 ± 0.01 | | 8.40 ± 0.03 | 7.34 ± 0.02 | 87.44 | 9.52 ± 0.02 | 8.20 ± 0.03 | 0.00 ± 0.00 | 0.00 | 9.07 ± 0.01 | 8.16 ± 0.02 | 8.90 ± 0.04 | 109.05 |  |
| *L. plantarum* A079 | 8.40 ± 0.10 | | 8.27 ± 0.04 | 8.03 ± 0.01 | 97.13 | 8.52 ± 0.02 | 8.47 ± 0.01 | 5.87 ± 0.02 | 69.36 | 8.48 ± 0.01 | 8.37 ± 0.02 | 7.60 ± 0.06 | 90.77 |  |
| *E. faecium* A080 | 9.08 ± 0.01 | | 8.50 ± 0.02 | 8.04 ± 0.00 | 94.62 | 9.41 ± 0.02 | 8.02 ± 0.02 | 5.33 ± 0.03 | 66.42 | 8.01 ± 0.01 | 8.08 ± 0.03 | 7.50 ± 0.13 | 92.81 |  |
| *E. lactis* A081 | 8.65 ± 0.03 | | 8.52 ± 0.05 | 7.95 ± 0.04 | 93.36 | 8.37 ± 0.02 | 8.23 ± 0.02 | 0.00 ± 0.00 | 0.00 | 8.12 ± 0.02 | 8.06 ± 0.05 | 7.88 ± 0.06 | 97.76 |  |
| *L. pseudomenseteroides* A082 | 8.52 ± 0.02 | | 8.37 ± 0.05 | 7.61 ± 0.01 | 90.88 | 8.42 ± 0.01 | 8.24 ± 0.03 | 5.34 ± 0.03 | 64.72 | 7.97 ± 0.02 | 7.63 ± 0.04 | 7.56 ± 0.05 | 99.13 |  |
| *E. faecium* A083 | 8.35 ± 0.10 | | 8.26 ± 0.06 | 7.76 ± 0.04 | 93.94 | 8.03 ± 0.02 | 7.95 ± 0.02 | 4.90 ± 0.06 | 61.66 | 8.17 ± 0.02 | 8.18 ± 0.02 | 7.92 ± 0.03 | 96.86 |  |
| *L. plantarum* A084 | 9.02 ± 0.02 | | 8.81 ± 0.04 | 8.60 ± 0.00 | 97.68 | 8.49 ± 0.01 | 8.42 ± 0.03 | 5.64 ± 0.03 | 67.00 | 8.34 ± 0.02 | 8.00 ± 0.03 | 7.77 ± 0.02 | 97.07 |  |
| *E. faecium* A087 | 8.53 ± 0.05 | | 7.97 ± 0.10 | 7.13 ± 0.01 | 89.44 | 8.51 ± 0.01 | 8.17 ± 0.02 | 0.00 ± 0.00 | 0.00 | 8.02 ± 0.02 | 8.01 ± 0.02 | 7.66 ± 0.05 | 95.59 |  |
| *L. pseudomenseteroides* A089 | 8.55 ± 0.05 | | 8.32 ± 0.04 | 8.07 ± 0.02 | 97.03 | 8.42 ± 0.01 | 7.98 ± 0.02 | 5.57 ± 0.03 | 69.78 | 8.30 ± 0.02 | 8.11 ± 0.03 | 6.77 ± 0.02 | 83.42 |  |
| *E. faecium* A090 | 8.46 ± 0.11 | | 8.04 ± 0.10 | 7.06 ± 0.02 | 87.77 | 8.32 ± 0.02 | 7.80 ± 0.06 | 0.00 ± 0.00 | 0.00 | 8.37 ± 0.59 | 7.40 ± 0.03 | 0.00 ± 0.00 | 0.00 |  |
| *L. pseudomenseteroides* A093 | 8.31 ± 0.06 | | 8.23 ± 0.04 | 7.13 ± 0.02 | 86.56 | 8.48 ± 0.02 | 7.88 ± 0.02 | 0.00 ± 0.00 | 0.00 | 7.86 ± 0.05 | 7.09 ± 0.03 | 7.70 ± 0.06 | 108.57 |  |
| *L. plantarum* A094 | 8.42 ± 0.18 | | 8.16 ± 0.03 | 6.99 ± 0.02 | 85.63 | 8.37 ± 0.01 | 8.28 ± 0.00 | 5.31 ± 0.03 | 64.20 | 8.17 ± 0.02 | 8.16 ± 0.02 | 6.85 ± 0.06 | 83.87 |  |
| *E. durans* A095 | 8.43 ± 0.09 | | 8.02 ± 0.10 | 8.00 ± 0.00 | 99.76 | 8.34 ± 0.02 | 8.32 ± 0.01 | 5.34 ± 0.05 | 64.18 | 8.49 ± 0.00 | 8.41 ± 0.06 | 8.22 ± 0.06 | 97.78 |  |

Mean±SD

Supplementary Table 2 cont’d

| LAB Isolates | pH3 (Log of cfu//ml) | | | | | pH2 (Log of cfu//ml) | | | | | 0.3 % Bile (Log of cfu//ml) | | | | | | | | |  |
| --- | --- | --- | --- | --- | --- | --- | --- | --- | --- | --- | --- | --- | --- | --- | --- | --- | --- | --- | --- | --- |
|  | Control | Initial | Final | Survival% | | Control | Initial | Final | Survival% | | | Control | | Initial | | Final | | Survival% | | |
| *E. lactis* A096 | 8.28 ± 0.13 | 8.18 ± 0.05 | 7.91 ± 0.02 | 96.72 | | 8.49 ± 0.01 | 8.08 ± 0.02 | 0.00 ± 0.00 | 0.00 | | | 8.18 ± 0.02 | | 8.14 ± 0.02 | | 7.12 ± 0.03 | | 87.46 | | |
| *E. durans* A097 | 9.04 ± 0.01 | 8.71 ± 0.03 | 7.53 ± 0.02 | 86.50 | | 8.31 ± 0.03 | 8.06 ± 0.02 | 5.48 ± 0.05 | 67.99 | | | 8.25 ± 0.02 | | 8.21 ± 0.01 | | 7.80 ± 0.02 | | 97.40 | | |
| *E. durans* A098 | 8.49 ± 0.15 | 8.00 ± 0.07 | 7.10 ± 0.02 | 88.77 | | 8.46 ± 0.01 | 8.41 ± 0.01 | 0.00 ± 0.00 | 0.00 | | | 8.02 ± 0.02 | | 7.40 ± 0.05 | | 0.00 ± 0.00 | | 0.00 | | |
| *E. faecalis* A4D | 8.43 ± 0.30 | 8.05 ± 0.07 | 8.00 ± 0.00 | 99.41 | | 8.56 ± 0.01 | 0.00 ± 0.00 | 0.00 ± 0.00 | 0.00 | | | 8.05 ± 0.03 | | 8.01 ± 0.02 | | 8.01 ± 0.03 | | 99.94 | | |
| *W. cibaria* B3b | 9.10 ± 0.03 | 8.47 ± 0.05 | 8.06 ± 0.02 | 95.19 | | 9.43 ± 0.01 | 8.36 ± 0.01 | 0.00 ± 0.00 | 0.00 | | | 8.46 ± 0.02 | | 8.17 ± 0.02 | | 7.59 ± 0.03 | | 92.92 | | |
| *L. pentosus* A1d | 8.52 ± 0.03 | 8.28 ± 0.08 | 7.86 ± 0.03 | 94.90 | | 8.39 ± 0.01 | 8.25 ± 0.01 | 5.33 ± 0.03 | 64.58 | | | 8.26 ± 0.01 | | 8.23 ± 0.01 | | 8.04 ± 0.03 | | 97.76 | | |
| *W. cibaria* B3a | 8.26 ± 0.14 | 8.09 ± 0.04 | 8.01 ± 0.00 | 98.91 | | 8.65 ± 0.01 | 8.27 ± 0.01 | 5.41 ± 0.03 | 65.40 | | | 8.11 ± 0.02 | | 8.11 ± 0.02 | | 8.10 ± 0.02 | | 99.86 | | |
| *L. fermentum* A3b | 8.23 ± 0.15 | 8.00 ± 0.06 | 7.78 ± 0.02 | 97.26 | | 8.04 ± 0.03 | 0.00 ± 0.00 | 0.00 ± 0.00 | 0.00 | | | 8.10 ± 0.01 | | 7.15 ± 0.11 | | 0.00 ± 0.00 | | 0.00 | | |
| *L. plantarum* B3c | 8.51 ± 0.05 | 8.19 ± 0.08 | 7.66 ± 0.03 | 93.59 | | 8.40 ± 0.01 | 8.26 ± 0.01 | 0.00 ± 0.00 | 0.00 | | | 8.18 ± 0.01 | | 8.15 ± 0.04 | | 8.14 ± 0.01 | | 99.91 | | |
| *L. plantarum* A4b | 8.58 ± 0.01 | 8.36 ± 0.09 | 7.80 ± 0.00 | 93.39 | | 8.21 ± 0.02 | 7.93 ± 0.02 | 5.05 ± 0.06 | 63.70 | | | 8.42 ± 0.00 | | 8.35 ± 0.02 | | 8.22 ± 0.04 | | 98.47 | | |
| *L. plantarum* B1b2 | 8.61 ± 0.01 | 8.38 ± 0.06 | 7.25 ± 0.01 | 86.48 | | 8.78 ± 0.00 | 8.51 ± 0.03 | 5.22 ± 0.07 | 61.30 | | | 8.36 ± 0.01 | | 8.36 ± 0.02 | | 8.22 ± 0.05 | | 98.38 | | |
| *L. plantarum* A1c | 8.58 ± 0.03 | 7.97 ± 0.09 | 7.54 ± 0.01 | 94.63 | | 9.53 ± 0.01 | 9.43 ± 0.03 | 5.99 ± 0.02 | 63.53 | | | 8.06 ± 0.01 | | 8.01 ± 0.02 | | 7.92 ± 0.10 | | 98.76 | | |
| *W. cibaria* B4a | 9.07 ± 0.01 | 8.26 ± 0.03 | 8.23 ± 0.00 | 99.73 | 9.38 ± 0.02 | | 8.18 ± 0.02 | 5.34 ± 0.03 | 65.25 | 8.26 ± 0.01 | | | 8.10 ± 0.05 | | 0.00 ± 0.00 | | 0.00 | |  |  |
| *L. pentosus* A4f | 8.56 ± 0.01 | 8.05 ± 0.07 | 7.62 ± 0.01 | 94.66 | 8.20 ± 0.02 | | 8.07 ± 0.02 | 0.00 ± 0.00 | 0.00 | 8.07 ± 0.01 | | | 8.02 ± 0.05 | | 8.01 ± 0.01 | | 99.94 | |  |  |
| *W. cibaria* B1e | 8.52 ± 0.05 | 8.20 ± 0.04 | 7.17 ± 0.02 | 87.38 | 8.73 ± 0.01 | | 8.23 ± 0.01 | 0.00 ± 0.00 | 0.00 | 8.22 ± 0.00 | | | 8.17 ± 0.01 | | 0.00 ± 0.00 | | 0.00 | |  |  |
| *L. fermentum* A3a | 8.32 ± 0.12 | 8.06 ± 0.07 | 8.03 ± 0.00 | 99.58 | 8.71 ± 0.01 | | 8.08 ± 0.04 | 0.00 ± 0.00 | 0.00 | 8.07 ± 0.00 | | | 7.99 ± 0.01 | | 0.00 ± 0.00 | | 0.00 | |  |  |
| *W. cibaria* B4c | 8.23 ± 0.15 | 8.00 ± 0.08 | 7.81 ± 0.00 | 97.69 | 8.42 ± 0.01 | | 8.25 ± 0.02 | 0.00 ± 0.00 | 0.00 | 8.00 ± 0.02 | | | 8.04 ± 0.04 | | 7.07 ± 0.02 | | 87.90 | |  |  |
| *L. pentosus* A4c | 8.43 ± 0.02 | 8.07 ± 0.06 | 8.06 ± 0.00 | 99.94 | 8.49 ± 0.01 | | 8.17 ± 0.01 | 5.83 ± 0.06 | 71.33 | 8.09 ± 0.04 | | | 8.04 ± 0.02 | | 8.02 ± 0.03 | | 99.76 | |  |  |
| *W. confuse* B1d | 8.53 ± 0.03 | 8.22 ± 0.06 | 7.07 ± 0.02 | 85.96 | 8.39 ± 0.06 | | 8.40 ± 0.04 | 0.00 ± 0.00 | 0.00 | 8.21 ± 0.02 | | | 8.10 ± 0.03 | | 7.58 ± 0.09 | | 93.52 | |  |  |
| *L. pentosus* A1a | 8.64 ± 0.05 | 8.35 ± 0.04 | 7.50 ± 0.01 | 89.81 | 8.69 ± 0.58 | | 8.15 ± 0.02 | 5.50 ± 0.02 | 67.42 | 8.35 ± 0.01 | | | 8.31 ± 0.01 | | 8.21 ± 0.05 | | 98.85 | |  |  |

Mean±SD

Supplementary Table 2 cont’d

| LAB Isolates | pH3 (Log of cfu//ml) | | | | | pH2 (Log of cfu//ml) | | | | | 0.3 % Bile (Log of cfu//ml) | | | | |
| --- | --- | --- | --- | --- | --- | --- | --- | --- | --- | --- | --- | --- | --- | --- | --- |
|  | Control | Initial | Final | Survival% | | Control | Initial | Final | Survival% | Control | | Initial | Final | Survival% |  |
| *L. plantarum* B1f | 8.50 ± 0.04 | 8.39 ± 0.08 | 8.07 ± 0.00 | 96.18 | 8.41 ± 0.05 | | 8.21 ± 0.02 | 0.00 ± 0.00 | 0.00 | 8.37 ± 0.00 | | 8.31 ± 0.01 | 8.00 ± 0.02 | 96.33 |  |
| *L. fermentum* A3d | 8.42 ± 0.07 | 8.24 ± 0.09 | 7.71 ± 0.00 | 93.60 | 8.27 ± 0.01 | | 8.07 ± 0.03 | 5.51 ± 0.03 | 68.34 | 8.20 ± 0.04 | | 8.14 ± 0.01 | 0.00 ± 0.00 | 0.00 |  |
| *L. plantarum* B1c | 8.49 ± 0.08 | 8.36 ± 0.03 | 8.39 ± 0.00 | 100.36 | 8.49 ± 0.01 | | 8.08 ± 0.04 | 0.00 ± 0.00 | 0.00 | 8.37 ± 0.02 | | 8.36 ± 0.02 | 8.29 ± 0.04 | 99.26 |  |
| *L. fermentum* A3c | 8.56 ± 0.07 | 8.19 ± 0.03 | 8.15 ± 0.00 | 99.55 | 8.16 ± 0.01 | | 8.12 ± 0.02 | 5.77 ± 0.02 | 71.11 | 8.21 ± 0.01 | | 8.37 ± 0.04 | 7.96 ± 0.02 | 95.03 |  |
| *L. pentosus* B1b | 8.63 ± 0.04 | 8.41 ± 0.06 | 8.37 ± 0.00 | 99.46 | | 8.48 ± 0.01 | 8.32 ± 0.01 | 5.88 ± 0.02 | 70.69 | 8.38 ± 0.02 | | 8.42 ± 0.00 | 8.30 ± 0.02 | 98.60 |  |
| *L. pentosus* A1e | 8.57 ± 0.01 | 8.32 ± 0.05 | 7.65 ± 0.01 | 91.91 | | 8.10 ± 0.01 | 7.72 ± 0.03 | 0.00 ± 0.00 | 0.00 | 8.31 ± 0.01 | | 8.25 ± 0.03 | 8.22 ± 0.03 | 99.71 |  |
| *L. plantarum* B1a | 8.53 ± 0.01 | 8.25 ± 0.02 | 8.24 ± 0.00 | 99.91 | | 8.74 ± 0.56 | 8.05 ± 0.03 | 5.73 ± 0.02 | 71.27 | 8.26 ± 0.02 | | 8.17 ± 0.01 | 8.20 ± 0.03 | 100.31 |  |
| *L. Xianqfangensis* B1a2 | 8.69 ± 0.03 | 8.42 ± 0.02 | 8.36 ± 0.00 | 99.21 | | 8.84 ± 0.56 | 8.47 ± 0.01 | 5.35 ± 0.02 | 63.17 | 8.43 ± 0.01 | | 8.42 ± 0.01 | 8.40 ± 0.01 | 99.86 |  |
| *E. durans* A004 | 8.48 ± 0.03 | 8.24 ± 0.04 | 8.02 ± 0.00 | 97.31 | | 8.03 ± 0.02 | 0.00 ± 0.00 | 0.00 ± 0.00 | 0.00 | 8.24 ± 0.02 | | 8.22 ± 0.02 | 7.86 ± 0.05 | 95.61 |  |
| *E. faecalis* A020 | 8.50 ± 0.03 | 8.34 ± 0.03 | 8.03 ± 0.00 | 96.35 | | 8.23 ± 0.02 | 8.20 ± 0.03 | 4.90 ± 0.06 | 59.80 | 8.37 ± 0.01 | | 8.20 ± 0.03 | 7.89 ± 0.08 | 96.26 |  |
| *E. faecalis* A029 | 8.43 ± 0.10 | 7.99 ± 0.05 | 8.08 ± 0.00 | 101.12 | | 8.07 ± 0.02 | 7.51 ± 0.03 | 0.00 ± 0.00 | 0.00 | 8.02 ± 0.02 | | 8.08 ± 0.02 | 7.95 ± 0.05 | 98.45 |  |
| *E. faecalis* A063 | 8.49 ± 0.03 | 8.05 ± 0.14 | 7.20 ± 0.01 | 89.40 | | 8.30 ± 0.02 | 8.18 ± 0.01 | 0.00 ± 0.00 | 0.00 | 8.00 ± 0.04 | | 8.01 ± 0.01 | 6.88 ± 0.06 | 85.95 |  |
| *E. faecalis* A078 | 8.72 ± 0.02 | 8.43 ± 0.09 | 8.42 ± 0.00 | 99.93 | | 8.52 ± 0.01 | 7.51 ± 0.07 | 0.00 ± 0.00 | 0.00 | 8.39 ± 0.03 | | 8.32 ± 0.01 | 6.69 ± 0.05 | 80.40 |  |

Mean±SD

**Supplementary Table 3. Affinity of lactic acid bacteria to n-hexadecane and p-xylene**

| LAB  Isolates | | n-Hexadecane | | | | Hydrophobicity % | | p-Xylene | | | | Hydrophobicity % | |
| --- | --- | --- | --- | --- | --- | --- | --- | --- | --- | --- | --- | --- | --- |
|  |  | Initial reading | | Final reading | | | | Initial reading | | Final reading | |  |  |
| *L. plantarum* A002 | | 0.78 ± 0.02 | | 0.57 ± 0.02 | | 26.21 | | 0.80 ± 0.01 | | 0.68 ± 0.00 | | 14.61 | |
| *E. faecium* A003 | | 0.77 ± 0.02 | | 0.74 ± 0.02 | | 4.49 | | 0.78 ± 0.01 | | 0.79± 0.00 | | -1.53 | |
| *E. faecium* A008 | | 0.72 ± 0.02 | | 0.72 ± 0.03 | | 0.05 | | 0.76 ± 0.01 | | 0.82 ± 0.00 | | -7.95 | |
| *L. pseudomesenteroides* A010 | | 0.78 ± 0.02 | | 0.75 ± 0.03 | | 3.76 | | 0.89 ± 0.01 | | 0.92 ± 0.00 | | -3.60 | |
| *L. plantarum* A011 | | 0.79 ± 0.02 | | 0.71 ± 0.03 | | 9.27 | | 0.81 ± 0.00 | | 0.70 ± 0.00 | | 14.41 | |
| *L. rhamnosus* A012 | | 0.81 ± 0.03 | | 0.61 ± 0.03 | | 25.01 | | 0.83 ± 0.01 | | 0.70 ± 0.00 | | 15.40 | |
| *E. durans* A013 | | 0.77 ± 0.02 | | 0.80 ± 0.03 | | -3.82 | | 0.78 ± 0.01 | | 0.80 ± 0.00 | | -2.010 | |
| *L. plantarum* A014 | | 0.86 ± 0.03 | | 0.78 ± 0.03 | | 8.95 | | 0.87 ± 0.00 | | 0.78 ± 0.00 | | 10.95 | |
| *L. pentosus* A016 | | 0.88 ± 0.01 | | 0.83 ± 0.01 | | 5.78 | | 0.88 ± 0.01 | | 0.83 ± 0.00 | | 5.06 | |
| *L. paracasei* A017 | | 0.78 ± 0.02 | | 0.59 ± 0.01 | | 25.17 | | 0.73 ± 0.00 | | 0.57 ± 0.00 | | 22.06 | |
| *E. faecium* A018 | | 0.75 ± 0.02 | | 0.77 ± 0.03 | | -1.91 | | 0.85 ± 0.01 | | 0.88 ± 0.01 | | -3.94 | |
| *L. pseudomesenteroides* A019 | | 0.70 ± 0.02 | | 0.57 ± 0.02 | | 19.40 | | 0.71 ± 0.00 | | 0.62 ± 0.00 | | 12.94 | |
| *E. faecium* A022 | | 0.76 ± 0.02 | | 0.81 ± 0.02 | | -5.45 | | 0.77 ± 0.00 | | 0.85 ± 0.02 | | -9.62 | |
| *L. plantarum* A023 | | 0.78 ± 0.02 | | 0.60 ± 0.02 | | 22.46 | | 0.79 ± 0.00 | | 0.72 ± 0.01 | | 9.95 | |
| *E. durans* A024 | | 0.79 ± 0.02 | | 0.79 ± 0.02 | | -0.34 | | 0.79 ± 0.00 | | 0.87 ± 0.00 | | -9.48 | |
| *L. pseudomesenteroides* A026 | | 0.70 ± 0.02 | | 0.40 ± 0.02 | | 42.13 | | 0.72 ± 0.00 | | 0.48 ± 0.00 | | 33.50 | |
| *E. thailandicus* A027 | | 0.72 ± 0.02 | | 0.68 ± 0.02 | | 5.48 | | 0.75 ± 0.01 | | 0.76 ± 0.00 | | -1.83 | |
| *L. pentosus* A028 | | 0.81 ± 0.02 | | 0.67 ± 0.02 | | 17.04 | | 0.83 ± 0.00 | | 0.82 ± 0.00 | | 0.52 | |
| *L. pseudomenseteroides* A030 | | 0.84 ± 0.02 | | 0.08 ± 0.00 | | 90.57 | | 0.85 ± 0.00 | | 0.24 ± 0.00 | | 72.09 | |
| *E. thailandicus* A031 | | 0.76 ± 0.02 | | 0.85 ± 0.01 | | -10.75 | | 0.77 ± 0.01 | | 0.97 ± 0.00 | | -25.84 | |
| *L. plantarum* A033 | | 0.76 ± 0.01 | | 0.74 ± 0.01 | | 3.10 | | 0.77 ± 0.01 | | 0.86 ± 0.00 | | -11.28 | |
| *L. plantarum A034* | | 0.90 ± 0.00 | | 0.81 ± 0.00 | | 9.65 | | 0.90 ± 0.00 | | 0.77 ± 0.00 | | 14.03 | |
| *L. plantarum* A035 | | 0.88 ± 0.02 | | 0.82 ± 0.02 | | 6.65 | | 0.89 ± 0.01 | | 0.89 ± 0.00 | | -0.56 | |
| *L. plantarum* A036 | | 0.85 ± 0.01 | | 0.91 ± 0.07 | | -7.21 | | 0.85 ± 0.00 | | 0.99 ± 0.01 | | -15.93 | |
| *L. plantarum* A037 | | 0.75 ± 0.00 | | 0.75 ± 0.02 | | 0.67 | | 0.75 ± 0.00 | | 0.74 ± 0.00 | | 1.82 | |
| *E. durans* A038 | | 0.78 ± 0.00 | | 0.81 ± 0.05 | | -3.86 | | 0.76 ± 0.00 | | 0.88 ± 0.00 | | -15.28 | |
| *E. faecium* A039 | | 0.70 ± 0.02 | | 0.66 ± 0.03 | | 5.89 | | 0.71 ± 0.00 | | 0.84 ± 0.00 | | -17.73 | |
| *E. faecium* A040 | | 0.71 ± 0.01 | | 0.74 ± 0.01 | | -3.99 | | 0.84 ± 0.00 | | 0.89 ± 0.00 | | -7.07 | |
| *L. plantarum* A041 | | 0.72 ± 0.00 | | 0.25 ± 0.00 | | 65.99 | | 0.73 ± 0.01 | | 0.67 ± 0.00 | | 8.88 | |
| *E. durans*A043 | | 0.79 ± 0.00 | | 0.78 ± 0.03 | | 1.73 | | 0.69 ± 0.01 | | 0.70 ± 0.00 | | -2.52 | |
| *L. pseudomenseteroides* A044 | | 0.79 ± 0.00 | | 0.71 ± 0.04 | | 10.46 | | 0.79 ± 0.00 | | 0.74 ± 0.00 | | 7.02 | |
| *L. plantarum* A046 | | 0.81 ± 0.01 | | 0.70 ± 0.03 | | 13.62 | | 0.81 ± 0.00 | | 0.89 ± 0.00 | | -9.95 | |

**Supplementary Table 3 cont’d**

| LAB Isolates | n-Hexadecane | | Hydrophobicity (%) | p-Xylene | | Hydrophobicity (%) |
| --- | --- | --- | --- | --- | --- | --- |
|  | Initial Reading | Final Reading |  | Initial Reading | Final Reading |  |
| *L. plantarum* A047 | 0.79 ± 0.00 | 0.70 ± 0.03 | 11.90 | 0.79± 0.00 | 0.77 ± 0.00 | 2.86 |
| *L. plantarum* A048 | 0.76 ± 0.00 | 0.72 ± 0.03 | 5.25 | 0.77 ± 0.01 | 0.74 ± 0.00 | 3.62 |
| *E. faecium* A049 | 0.74 ± 0.00 | 0.78 ± 0.03 | -6.11 | 0.63 ± 0.01 | 0.64 ± 0.00 | -1.59 |
| *E. durans* A050 | 0.71 ± 0.01 | 0.71 ± 0.02 | -0.28 | 0.70 ± 0.00 | 0.88 ± 0.00 | -25.75 |
| *L. plantarum* A051 | 0.71 ± 0.01 | 0.66 ± 0.01 | 7.53 | 0.72 ± 0.00 | 0.64 ± 0.00 | 10.74 |
| *E. lactis* A052 | 0.74 ± 0.00 | 0.62 ± 0.01 | 16.85 | 0.77 ± 0.00 | 0.47 ± 0.00 | 38.72 |
| *E. faecalis* A058 | 0.76 ± 0.00 | 0.78 ± 0.03 | -3.26 | 0.77 ± 0.00 | 0.85 ± 0.00 | -10.50 |
| *L. plantarum* A059 | 0.89 ± 0.01 | 0.89 ± 0.02 | -0.49 | 0.87 ± 0.00 | 0.93 ± 0.02 | -6.91 |
| *E. lactis* A060 | 0.77 ± 0.01 | 0.77 ± 0.02 | -0.43 | 0.88 ± 0.00 | 0.53 ± 0.00 | 32.75 |
| *L. pseudomenseteroides*A064 | 0.76 ± 0.00 | 0.56 ± 0.02 | 26.95 | 0.77 ± 0.00 | 0.77 ± 0.00 | 0.65 |
| *E. faecium* A066 | 0.76 ± 0.02 | 0.83 ± 0.09 | -9.43 | 0.81 ± 0.00 | 0.88 ± 0.00 | -8.23 |
| *L. plantarum* A071 | 0.86 ± 0.04 | 0.87 ± 0.02 | -1.94 | 0.72 ± 0.00 | 0.65 ± 0.00 | 9.59 |
| *L. rhamnosus* A072 | 0.71 ± 0.00 | 0.63 ± 0.00 | 11.63 | 0.71 ± 0.00 | 0.65 ± 0.00 | 8.01 |
| *P. pentosaceus* A074 | 0.79 ± 0.00 | 0.71 ± 0.02 | 10.66 | 0.79 ± 0.00 | 0.78 ± 0.00 | 1.39 |
| *L. plantarum* A075 | 0.71 ± 0.00 | 0.50 ± 0.00 | 29.61 | 0.72 ± 0.00 | 0.63 ± 0.00 | 12.62 |
| *E. faecalis* A077 | 0.76 ± 0.03 | 0.73 ± 0.02 | 4.29 | 0.83 ± 0.00 | 0.83 ± 0.00 | 0.08 |
| *L. plantarum* A079 | 0.85 ± 0.02 | 0.83 ± 0.02 | 2.73 | 0.86 ± 0.00 | 0.89 ± 0.00 | -3.40 |

**Supplementary Table 3 cont’d**

| LAB Isolates | n-Hexadecane | | Hydrophobicity (%) | p-Xylene | | | Hydrophobicity (%) |
| --- | --- | --- | --- | --- | --- | --- | --- |
|  | Initial reading | Final reading |  | Initial reading | | Final reading |  |
| *E. faecium* A080 | 0.73 ± 0.01 | 0.72 ± 0.01 | 2.17 | 0.79 ± 0.01 | 0.81 ± 0.00 | | -2.74 |
| *E. lactis* A081 | 0.78 ± 0.01 | 0.78 ± 0.02 | 0.13 | 0.79 ± 0.00 | 0.79 ± 0.00 | | -0.59 |
| *L. pseudomenseteroides*A082 | 0.87 ± 0.01 | 0.72 ± 0.01 | 18.03 | 0.71 ± 0.00 | 0.60 ± 0.00 | | 15.20 |
| *E. faecium* A083 | 0.76 ± 0.00 | 0.76 ± 0.02 | 0.39 | 0.75 ± 0.00 | 0.73 ± 0.00 | | 2.83 |
| *L. plantarum* A084 | 0.71 ± 0.00 | 0.71 ± 0.00 | -0.24 | 0.71 ± 0.00 | 0.70 ± 0.00 | | 1.12 |
| *E. faecium* A087 | 0.71 ± 0.00 | 0.17 ± 0.00 | 75.80 | 0.70 ± 0.00 | 0.76 ± 0.00 | | -7.69 |
| *L.. pseudomenseteroides* A089 | 0.70 ± 0.00 | 0.69 ± 0.00 | 1.24 | 0.71 ± 0.00 | 0.69 ± 0.00 | | 3.34 |
| *E. faecium A090* | 0.70 ± 0.00 | 0.58 ± 0.03 | 17.21 | 0.72 ± 0.00 | 0.55 ± 0.00 | | 23.47 |
| *L. pseudomesenteroides* A093 | 0.77 ± 0.00 | 0.64 ± 0.01 | 17.34 | 0.75 ± 0.00 | 0.51 ± 0.00 | | 32.83 |
| *L. plantarum* A094 | 0.79 ± 0.00 | 0.81 ± 0.01 | -3.40 | 0.84 ± 0.00 | 0.88 ± 0.00 | | -4.12 |
| *E. durans* A095 | 0.71 ± 0.00 | 0.66 ± 0.00 | 5.95 | 0.77 ± 0.00 | 0.81 ± 0.00 | | -5.22 |
| *E. lactis* A096 | 0.78 ± 0.01 | 0.77 ± 0.01 | 1.53 | 0.72 ± 0.00 | 0.78 ± 0.00 | | -8.43 |
| *E. durans* A097 | 0.74 ± 0.00 | 0.73 ± 0.03 | 1.63 | 0.74 ± 0.01 | 0.73 ± 0.00 | | 0.59 |
| *E. durans* A098 | 0.86 ± 0.00 | 0.85 ± 0.02 | 0.70 | 0.71 ± 0.00 | 0.77 ± 0.02 | | -7.92 |
| *E. faecalis* A4D | 0.79 ± 0.00 | 0.77 ± 0.02 | 1.82 | 0.71 ± 0.00 | 0.80 ± 0.00 | | -13.81 |
| *W. cibaria* B3b | 0.75 ± 0.00 | 0.61 ± 0.01 | 18.71 | 0.76 ± 0.01 | 0.7 2± 0.00 | | 5.14 |
| *L. pentosus* A1d | 0.76 ± 0.00 | 0.72 ± 0.01 | 4.97 | 0.78 ± 0.00 | 0.76 ± 0.00 | | 2.32 |

**Supplementary Table 3 cont’d**

| LAB Isolates | n-Hexadecane | | Hydrophobicity (%) | p-Xylene | | | Hydrophobicity (%) |
| --- | --- | --- | --- | --- | --- | --- | --- |
|  | Initial reading | Final reading |  | Initial reading | | Final reading |  |
| *W. cibaria* B3a | 0.82 ± 0.00 | 0.69 ± 0.05 | 15.22 | 0.89±0.00 | 0.85 ± 0.00 | | 4.63 |
| *L. fermentum* A3b | 0.85 ± 0.02 | 0.56 ± 0.01 | 34.25 | 0.74 ±0.00 | 0.80 ± 0.00 | | -8.87 |
| *L. plantarum* B3c | 0.73 ± 0.00 | 0.69 ± 0.02 | 5.91 | 0.73 ±0.00 | 0.71 ± 0.00 | | 2.66 |
| *L. plantarum* A4b | 0.73 ± 0.01 | 0.52 ± 0.01 | 28.64 | 0.75 ±0.01 | 0.59 ± 0.00 | | 20.78 |
| *L. plantarum* B1b2 | 0.87 ± 0.02 | 0.97 ± 0.02 | -11.57 | 0.82±0.00 | 0.86 ± 0.00 | | -4.09 |
| *L. plantarum* A1c | 0.85 ± 0.02 | 1.04 ± 0.03 | -22.43 | 0.75 ±0.00 | 0.70 ± 0.00 | | 6.39 |
| *W. cibaria*B4a | 0.77 ± 0.02 | 0.82 ± 0.02 | -6.21 | 0.88±0.00 | 0.86 ± 0.00 | | 2.38 |
| *L. pentosus*A4f | 0.88 ± 0.02 | 0.90 ± 0.02 | -1.62 | 0.71±0.00 | 0.89 ± 0.00 | | -25.15 |
| *W. cibaria*B1e | 0.89 ± 0.01 | 0.96 ± 0.02 | -8.02 | 0.87±0.00 | 1.13 ± 0.00 | | -29.20 |
| *L. fermentum* A3a | 0.78 ± 0.00 | 0.62 ± 0.03 | 20.89 | 0.75 ±0.00 | 0.63 ± 0.00 | | 16.09 |
| *W. cibaria*B4c | 0.73 ± 0.01 | 0.74 ± 0.03 | -1.93 | 0.72 ±0.00 | 0.83 ± 0.00 | | -15.50 |
| *L. pentosus* A4c | 0.87 ± 0.03 | 0.78 ± 0.03 | 11.03 | 0.80 ±0.00 | 0.67 ± 0.00 | | 16.01 |
| *W. confusa*B1d | 0.71 ± 0.00 | 0.64 ± 0.03 | 9.74 | 0.90 ±0.00 | 0.82 ± 0.00 | | 8.27 |
| *L. pentosus* A1a | 0.82 ± 0.00 | 0.73 ± 0.03 | 10.93 | 0.89 ±0.00 | 0.89 ± 0.00 | | -0.34 |
| *L. plantarum* B1f | 0.74 ± 0.01 | 0.73 ± 0.01 | 1.17 | 0.71±0.00 | 0.75 ± 0.00 | | -5.14 |
| *L. fermentum* A3d | 0.75 ± 0.00 | 0.72 ± 0.00 | 3.21 | 0.79 ±0.00 | 0.76 ± 0.00 | | 3.76 |
| *L. plantarum* B1c | 0.79 ± 0.01 | 0.65 ± 0.03 | 17.94 | 0.76±0.00 | 0.73 ± 0.00 | | 5.10 |
| *L. fermentum* A3c | 0.82 ± 0.00 | 0.83 ± 0.02 | -0.93 | 0.90 ±0.00 | 0.90 ± 0.00 | | -0.74 |

**Supplementary Table 3 cont’d**

| LAB Isolates | n-Hexadecane | | Hydrophobicity (%) | p-Xylene | | Hydrophobicity (%) |
| --- | --- | --- | --- | --- | --- | --- |
|  | Initial Reading | Final Reading |  | Initial Reading | Final Reading |  |
| *L. pentosus* B1b | 0.79 ± 0.01 | 0.74 ± 0.01 | 7.27 | 0.86 ± 0.00 | 0.85 ± 0.00 | 0.59 |
| *L. pentosus* A1e | 0.79 ± 0.02 | 0.0 ± 0.01 | 24.31 | 0.70 ± 0.00 | 0.75 ± 0.00 | -7.20 |
| *L. plantarum* B1a | 0.85 ± 0.01 | 0.74 ± 0.01 | 12.72 | 0.86 ± 0.00 | 0.79 ± 0.00 | 9.14 |
| *L. xianqfangensis* B1a2 | 0.66 ± 0.11 | 0.57 ± 0.10 | 13.75 | 0.79 ± 0.01 | 0.76 ± 0.00 | 3.44 |
| *E. durans*A004 | 0.73 ± 0.01 | 0.74 ± 0.01 | -1.00 | 0.73 ± 0.00 | 0.71 ± 0.00 | 2.25 |
| *E. faecalis* A020 | 0.68 ± 0.10 | 0.58 ± 0.00 | 15.36 | 0.72 ± 0.01 | 0.62 ± 0.00 | 13.10 |
| *E. faecalis* A029 | 0.82 ± 0.05 | 0.79 ± 0.06 | 3.90 | 0.87 ± 0.00 | 0.91 ± 0.06 | -3.70 |
| *E. faecalis* A063 | 0.79 ± 0.00 | 1.04 ± 0.02 | -32.34 | 0.85 ± 0.00 | 0.99 ± 0.00 | -15.38 |
| *E. faecalis A078* | 0.75 ± 0.01 | 0.71 ± 0.00 | 5.62 | 0.76 ± 0.00 | 0.83 ± 0.00 | -8.70 |
